# Dinuclear Copper Sulfate-Based Square Lattice Topology Network with High Alkyne Selectivity

**DOI:** 10.1021/acs.cgd.4c00094

**Published:** 2024-02-27

**Authors:** Yassin H. Andaloussi, Debobroto Sensharma, Andrey A. Bezrukov, Dominic C. Castell, Tao He, Shaza Darwish, Michael J. Zaworotko

**Affiliations:** Department of Chemical Sciences, Bernal Institute, University of Limerick, Limerick V94 T9PX, Republic of Ireland

## Abstract

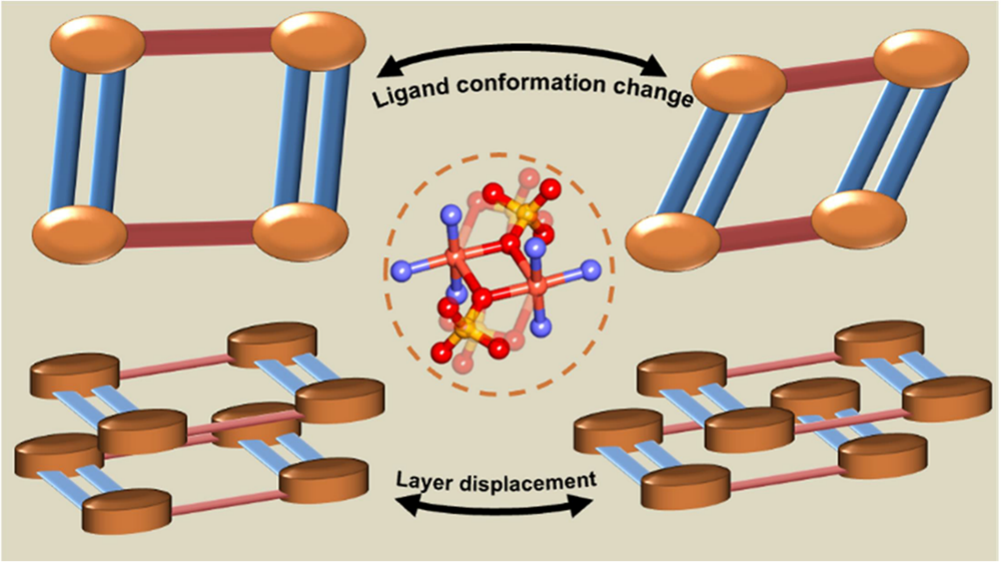

Porous coordination networks (PCNs) sustained by inorganic anions that serve as linker ligands can offer high selectivity toward specific gases or vapors in gas mixtures. Such inorganic anions are best exemplified by electron-rich fluorinated anions, e.g., SiF_6_^2–^, TiF_6_^2–^, and NbOF_5_^2–^, although sulfate anions have recently been highlighted as inexpensive and earth-friendly alternatives. Herein, we report the use of a rare copper sulfate dimer molecular building block to generate two square lattice, **sql**, coordination networks which can be prepared via solvent layering or slurrying, CuSO_4_(1,4-bib)_1.5_, **1**, (1,4-bib = 1,4-bisimidazole benzene) and CuSO_4_(1,4-bin)_1.5_, **2**, (1,4-bin = 1,4-bisimidazole naphthalene). Variable-temperature SCXRD and PXRD experiments revealed that both **sql** networks underwent reversible structural transformations due to linker rotations or internetwork displacements. Gas sorption studies conducted upon the narrow-pore phase of CuSO_4_(1,4-bin)_1.5_, **2np**, found a high calculated 1:99 selectivity for C_2_H_2_ over C_2_H_4_ (33.01) and CO_2_ (15.18), as well as strong breakthrough performance. Across-the-board, C_3_H_4_ selectivity vs C_3_H_6_, CO_2_, and C_3_H_8_ was also observed. Sulfate-based PCNs, although still understudied, appear increasingly likely to offer utility in gas and vapor separations.

## Abstract 2

Two square lattice **sql** coordination networks, CuSO_4_(1,4-bib)_1.5_ and CuSO_4_(1,4-bin)_1.5_, formed from copper sulfate molecular building blocks undergo reversible structural transformation due to linker rotations or layer displacements, allowing for stepped gas sorption isotherms.

## 1 Introduction

Porous coordination networks (PCNs)^1,2^ are a class of sorbents that are sustained by inorganic and/or organic ligands that link metal ions to form porous networks. The properties of PCNs offer promise for purification or storage of commodities such as carbon dioxide, methane,^3,4^ water vapor,^5−7^ and hydrocarbon gas mixtures.^8,9^ PCNs are also of interest for the sensing and removal of trace contaminants such as heavy metals^10^ or volatile organic compounds (VOCs).^11,12^ Alkene/alkane separations have been listed as one of the “seven chemical separations to change the world”.^13^ However, few sorbents of any type, even if they exhibit high capacity, high selectivity, and fast kinetics, can also meet the requirements of being cost-effective, stable, regenerable, and readily scalable.^14^

Whereas most PCNs are comprised of metal ions or metal clusters and organic linker ligands, which would classify them as metal–organic frameworks, MOFs, there are also PCNs based upon purely inorganic linker ligands, e.g., Prussian blue,^15^ or mixtures of inorganic and organic linker ligands, e.g., hybrid ultramicroporous materials, HUMs.^16^ HUMs are exemplified by hexafluorosilicate (SIFSIX) PCNs such as the microporous PCNs SIFSIX-1-Zn and SIFSIX-1-Cu (1 = 4,4′-bipyridine), reported in 1995^17^ and 2000,^18^ respectively. HUMs have since been expanded to include a range of fluorinated inorganic anions^19,20^ comprising metal centers such as Ti,^21,22^ Nb,^23^ Ge,^24^ Zr,^25^ Al,^26^ and Ta.^27^ Such sorbents are of particular note because they can exhibit exceptional trace gas separation properties.^28,29^ Unfortunately, the use of fluoride-based anions can be a cause for concern due to the risk of HF exposure during inorganic anion synthesis, or upon thermal decomposition of the PCN,^30^ or otherwise pose health and environmental risks due to the use of toxic heavy metal ions. As such, the use of the more earth-friendly and abundant sulfate (SOFOUR) anion has recently been explored by us, e.g., SOFOUR-1-Zn,^31^ and others, e.g., SOFOUR-TEPE-Zn,^32^ for the trace separation of C_2_H_2_ from CO_2_.

**Scheme 1:**
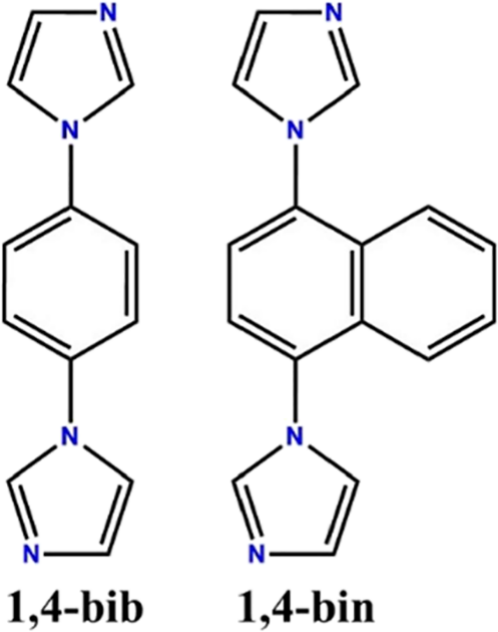
1,4-Bisimidazole Benzene (1,4-Bib) (Left) and 1,4-Bisimidazole Naphthalene (1,4-Bin) (Right)

**Figure 1:**
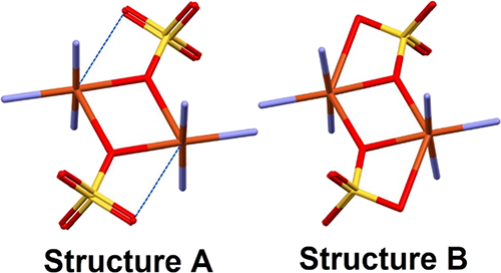
Structural variation in the CuSO_4_ MBB dimer with Structure A characterized by (μ_2_-η^2^) monatomic bridging groups or Structure B with (μ_2_-η:^2^η^1^) monatomic bridging sulfate groups with chelation to the copper open metal site.

In this study, we explore the structure and sorption properties of square lattice, **sql**, topology networks involving sulfate anions following a report by Xie et al.^33^ on the product formed by reacting copper sulfate and 1,4-bisimidazole benzene (1,4-bib), CuSO_4_(1,4-bib)_1.5_, **1**, which exhibits an uncommon copper sulfate dimer molecular building block (MBB). We detail the synthesis, by both solvent layering and slurrying, and sorption properties of **1** and its isoreticular analog CuSO_4_(1,4-bin)_1.5_, **2**, (1,4-bin = 1,4,-bisimidazolenaphthalene) (Scheme 1), with each exhibiting different sorption behavior. A Cambridge Structural Database (CSD)^34^ analysis of the copper sulfate MBB revealed only 24 hits (Table S1) with two structural variants, A and B (Figure 1), involving an additional interaction between sulfate oxygens and the copper open metal site. The structural variants are characterized by a bimodal distribution in the lengths of this additional interaction, with distances ranging between 2.6 and 2.9 and 3.0–3.3 Å (Figure S4). Among the structures that form this MBB, the majority are 0D compounds with only 6 forming coordination polymers.^33,35−39^ Study of the gas sorption properties in this family of materials has not been reported, a matter that is addressed herein.

## 2 Results and Discussion

### 2.1 CuSO_4_(1,4-bib)_1.5_, **1**

**Figure 2:**
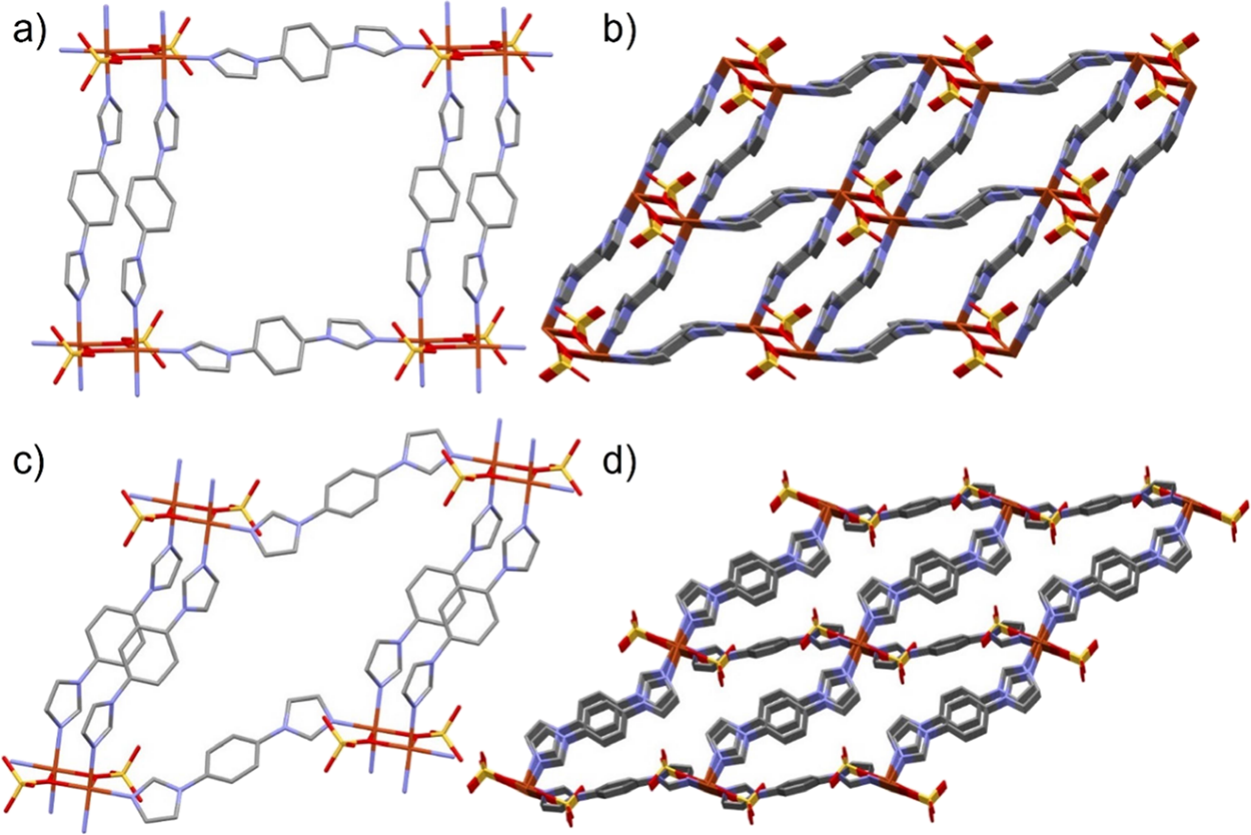
(a) **sql** net of **1op** at 100 K formed from single-ligand and double-ligand walls; (b) **1op** at 100 K viewed along the *c*-axis; (c) **sql** net of **1cp** at 343 K; and (d) **1cp** at 343 K viewed along the *b*-axis. Hydrogen atoms have been omitted for clarity.

MeOH/H_2_O layering, or the more readily scalable method of slurrying, was employed to prepare **1**. Unless otherwise stated, **1** synthesized through layering was used for all further characterization. As reported by Xie et al.,^33^**1** crystallized in the triclinic P1̅ space group (Table S3). **1** is sustained by the Structure A variant of the copper sulfate dimer MBB (Figure 1). The two copper ions are bridged by two sulfate ions that bond in a monatomic bridging (μ_2_-η_2_)-fashion with bond lengths of 2.0107(12) and 2.3302(12) Å to generate a Cu_2_O_2_ ring. Each copper ion exhibits a square pyramidal coordination environment with bonds to imidazole groups of 1,4-bib above and below the Cu_2_O_2_ plane with Cu–N bond lengths 1.9870(13) and 1.9865(13) Å. A third 1,4-bib binds in a coplanar manner to the Cu_2_O_2_ plane with a Cu–N bond length of 2.0287(14) Å (Figure 2a, Table S2). This leaves the base of the square pyramidal Cu ion as an open metal site, with the nearest sulfate oxygen at a nonbonding distance of 3.0503(17) Å (Structure A, Figure 1). While the CuSO_4_ MBB dimer binds 6 imidazole ligands in total, two pairs of ligands bind in parallel orientations above and below the Cu_2_O_2_ plane, forming a double-ligand “wall” to the next MBB. This results in the MBB acting as a 4-connected node that links through the double-ligand wall and, via the coplanar 1,4-bib ligand, single-ligand walls to form a 2D square lattice (**sql**) topology (Figure 2a). The **sql** layers stack, resulting in a void space of 12.7% thanks to 1D pores that are aligned parallel to the *c*-axis (Figure 2b).

Water molecules lie in the pores with a crystallographically determined water content of 2.45 molecules per formula unit (resolved at 100 K). One water molecule forms hydrogen bonds with two separate sulfate ions O–H–O bond lengths of 2.766(3) and 2.825(4) Å (Table S2). As observed in successive SCXRD experiments conducted at RT and 333 K, this water molecule persisted in the structure up to 333 K while the other water molecules desorbed under these conditions. These structural results are consistent with thermogravimetric analysis (TGA) (Figure S6) and differential scanning calorimetry (DSC) (Figure S6) results, each of which revealed two thermal events. Further heating to 343 K in situ on the SCXRD goniometer was found to induce a phase transition to a closed-pore (**1cp**) structure concomitant with the removal of the strongly bound water molecule. SCXRD data revealed that **1cp** retained the connectivity and topology of the open phase, **1op**, with the unit cell volume decreasing by 5% (Figure S7, Table S3). Conformational changes in the single-wall 1,4-bib ligand (Figures 2c and S8) resulted in the square grid net contorting, with the Cu–Cu–Cu angle in the **sql** net rising from 101.494(4)° in **1op** to 125.809(11)° in **1cp**. The 1,4-bib imidazole angle with the Cu_2_O_2_ plane increased from 28.18(7)° in **1op** at 100 K to 50.0(3)° in **1cp** at 333 K, with the void space dropping to 3.9% of the unit cell in the form of isolated cavities (Figure 2d, Table S2). More details on the structural parameters of **1** at 100, 299, 333, and 343 K are given in Table S2.

As shown by VTPXRD experiments under N_2_ flow (Figure S9), **1op** powder exhibited this phase transition at ca. 373 K, with full conversion to **1cp** observed at 413 K. **1cp** persisted after cooling to 293 K under nitrogen flow; however, exposure to air with ambient humidity for 5 min caused the sample to revert to **1op**. These observations are consistent with dynamic vapor sorption (DVS) experiments, which revealed that water vapor uptake occurred at very low RH (<2%) for uptake of 10.5 weight% at 298 K. A step in the isotherm was observed at ca. 5% RH at 333 K (Figures S10 and S11).

**Figure 3:**
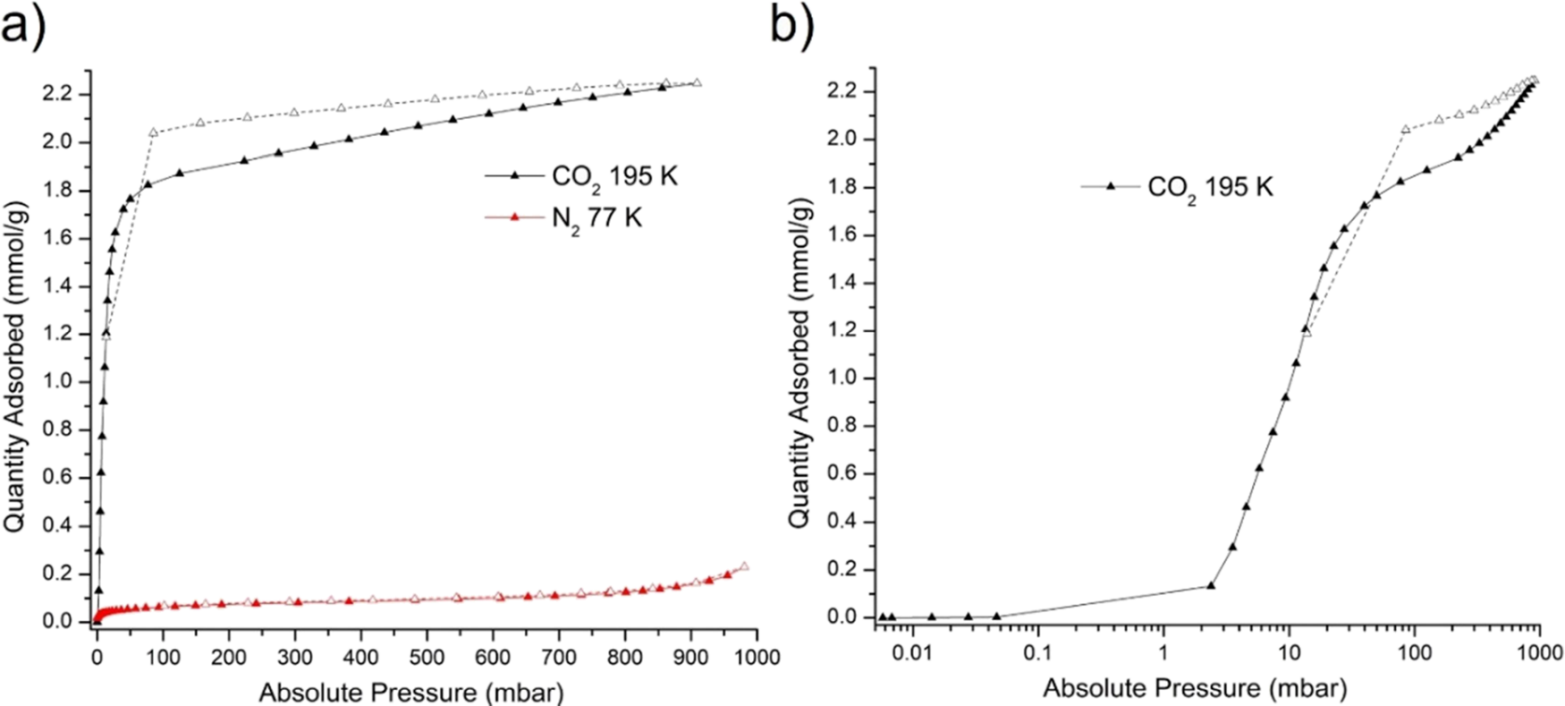
(a) Low pressure gas sorption of **1** for CO_2_ at 195 K and N_2_ at 77 K. (b) Low pressure gas sorption of **1** for CO_2_ at 195 K on a logarithmic pressure scale.

The gas sorption properties of **1** were studied for N_2_ at 77 K and CO_2_ at 195 K using a sample evacuated at 373 K to produce **1cp**, for which it was found that no appreciable uptake occurred for N_2_ while CO_2_ adsorbed 2.25 mmol/g in an apparent type I isotherm (Figure 3a). Closer inspection with pressure plotted on a logarithmic scale revealed this to be an isotherm with a gate-opening pressure observed at ca. 2 mbar (Figure 3b). No appreciable uptake was observed for low pressure isotherms of CO_2_ at 298 K, whereas high-pressure CO_2_ data collected at 273, 298, 303, 308, and 313 K afforded isotherms resembling that for 195 K CO_2_ (Figure S12). Each isotherm displayed an initially concave adsorption profile corresponding to a type F–III isotherm.^40^ Low pressure gas sorption isotherms collected at 298 K for C2 hydrocarbons showed no uptake (Figure S13). 298 K CO_2_ adsorption isotherms were also measured for a RT-activated sample of CuSO_4_(1,4-bib)_1.5_ to determine whether **1op** had differing sorption properties,; however, subsequent cycles showed diminishing uptake due to a slow **1op**–**1cp** phase transition under vacuum at RT (Figure S14).

### 2.2 CuSO_4_(1,4-bin)_1.5_, **2**

**Figure 4:**
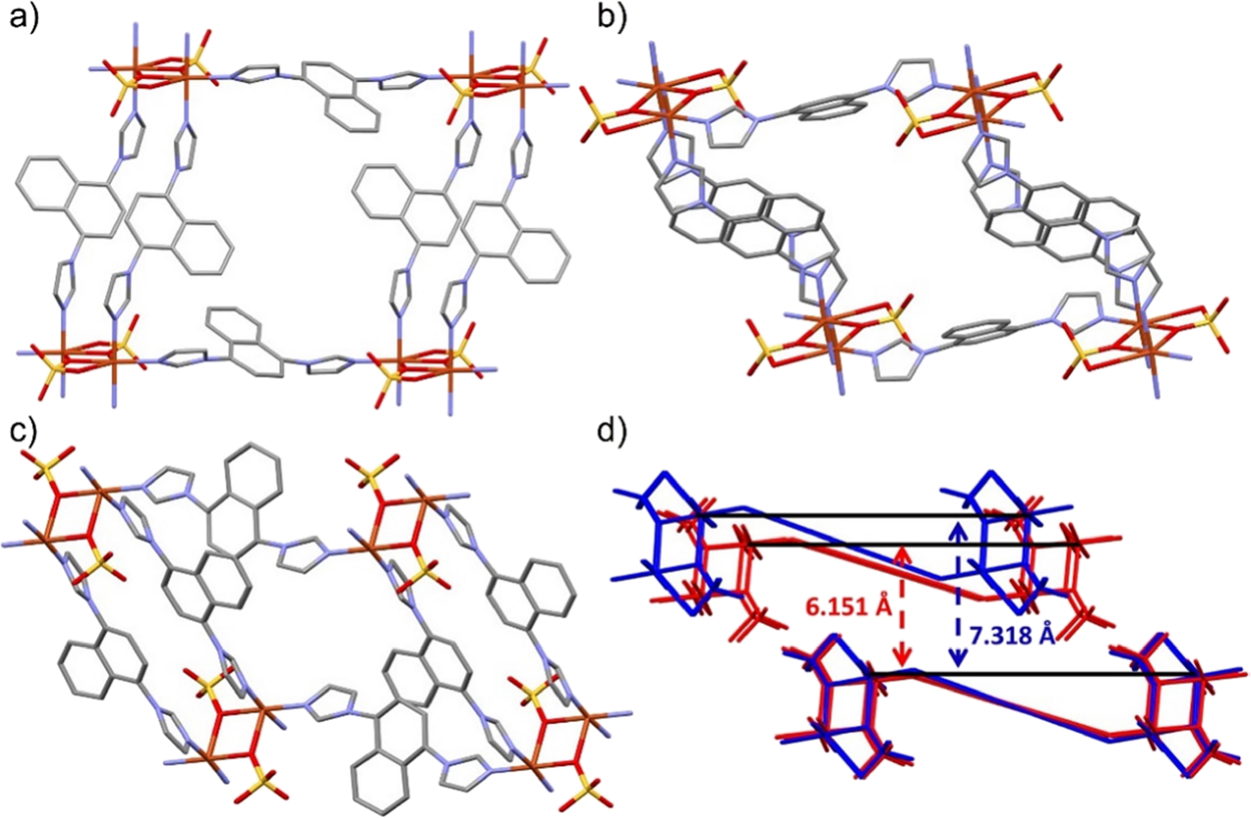
(a) Portion of the **sql** net of **2op** at 100 K; (b) view along the *a*-axis for **2op**; (c) view along the *a*-axis for **2np** at 100 K. Disordered groups and hydrogen atoms have been omitted for clarity; (d) overlay of **2op** (blue) and **2np** (red) with the interlayer distance between corresponding copper atoms. Overlay of Cu_2_O_2_ moieties with 1,4-bin ligands was replaced with a single bond for clarity.

Given the structural features and switching gas sorption behavior of **1**, the bulkier naphthalene variant of 1,4-bib was also investigated. When 1,4-bisimidazole naphthalene (1,4-bin) was layered with copper sulfate in H_2_O/(CH_2_OH)_2_/MeOH, **2** was formed. **2** was also subsequently produced through slurrying in MeOH. Unless otherwise stated, **2** synthesized through layering was used for further characterization. As with **1**, **2** crystallized in the triclinic space group P1̅ and formed the CuSO_4_ MBB dimer with Cu–O bond lengths of 2.0157(15) and 2.3202(13) Å in the Cu_2_O_2_ ring, when measured at 100 K (Tables S4 and S6). **2** exhibited the “Structure B” MBB variant with a Cu–O distance of 2.7607(17) Å (Figure 1), resulting in (μ_2_-η:^2^η^1^) monatomic bridging and chelation. The Cu ions exhibit distorted octahedral coordination (Table S4). Disorder, however, was evident in sulfate ligands consistent with a minor disordered component (with freely refined occupancy of ca. 14.5%) of the “Structure A” MBB variant with a Cu–O distance of 3.170(15) Å (Figure S1 and Table S4). As with **1**, **2** formed double-ligand and single-ligand walls, resulting in **sql** topology (Figure 4a). Within the single-ligand wall, the naphthalene moiety was found to be disordered about a center of inversion. 23.9% porosity was calculated from pores along the a- and b-axes (Figure 4b) and a 7.318 Å interlayer spacing between **sql** sheets (Figure 4d). O–O distances between sulfate groups are 6.791(5), 8.572(6), and 8.669(5) Å.

Removal of solvent molecules from the as-synthesized phase, **2op**, by exposure to low RH air, vacuum, or heat, resulted in formation of a narrow-pore phase, **2np**, which also crystallized in the triclinic space group P1̅ with a 16.5% lower unit cell volume than **2op**, when measured at 100 K. Unlike **1**, solvent removal did not lead to conformational variation between **2op** and **2np** (Figure S17). There was, however, elongation of the copper sulfate interaction to 2.837(12) Å. Additionally, a reduced torsion angle was observed between the single-ligand wall imidazole group and the Cu_2_O_2_ ring, from 27.57(7)° in **2op** to 18.68(19)° in **2np**. Disorder in **2np** was observed in the naphthalene moiety, disordered around a center of inversion, and also in the imidazole and sulfate groups (Figure S2). The minor components, refined at a fixed 25% occupancy, display an imidazole group rotated by 88.46(17)° and a sulfate group with diatomic (μ_2_-η:^1^η^1^) bridging. **2np** was calculated to have 11.3% porosity through 1D continuous channels (Figure 4c) and a reduced interlayer spacing of 6.151 Å (Figure 4d). The O–Å distance between sulfate groups is 8.075(17) Å. Additional structural parameters for **2** at 100 K and RT are detailed in Tables S4 and S5.

**Figure 5:**
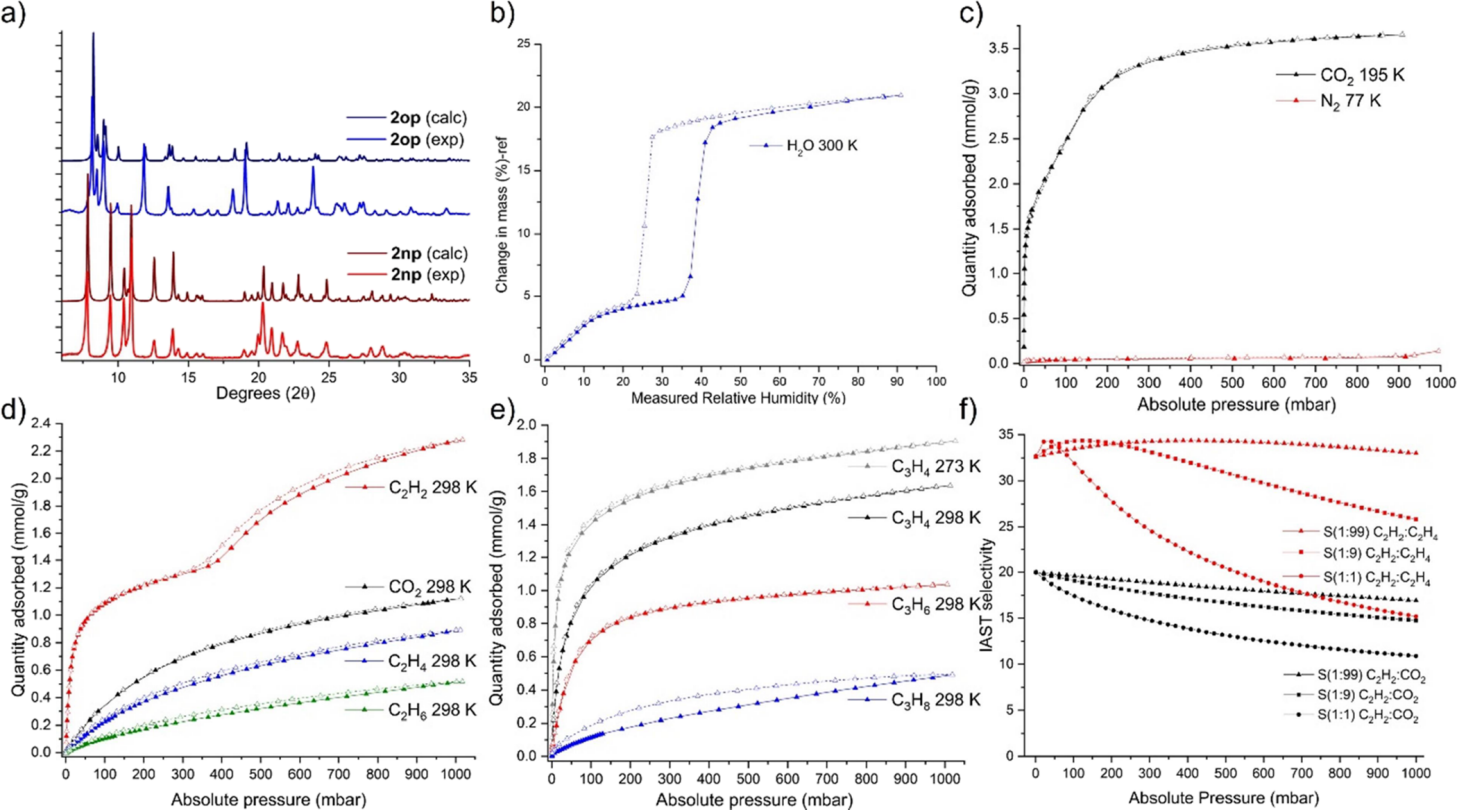
(a) Overlay of calculated (at RT) and experimental PXRD patterns of **2op** and **2np** synthesized by layering; (b) water vapor sorption of **2** at 300 K; (c) CO_2_ and N_2_ adsorption isotherms of **2** at 195 and 77 K, respectively; (d) CO_2_, C_2_H_2_, C_2_H_4_, and C_2_H_6_ adsorption isotherms of **2** at 298 K; (e) C_3_H_4_ adsorption isotherms of **2** at 273 and 298 K and C_3_H_6_ and C_3_H_8_ at 298 K; (f) C_2_H_2_:CO_2_ and C_2_H_2_:C_2_H_4_ IAST selectivity of **2** at 298 K for various compositions vs pressure.

The transformation from **2op** to **2np** was studied through PXRD (Figure 5a), VTPXRD (Figure S20), TGA (Figure S18), and DSC (Figure S19). In DVS experiments (Figure 5b), **2np** was found to convert to **2op** reversibly, as indicated by a type F–II isotherm profile, i.e., gradual uptake of 4.7 wt % up to 34% RH, followed by sharp uptake characteristic of a phase transition at 39% RH, for a total uptake of 22.0 wt %. Water vapor desorption occurred with hysteresis at 26% RH, indicating reversion to **2np**. Similar properties were observed for a sample synthesized through slurrying, however, with a larger hysteresis and **2op** being formed at 50% RH (Figure S19b). These differences could be attributed to smaller particle size as evidenced by the comparatively broader PXRD of the slurry-based product (Figure S19a).

The porosity of **2** was investigated for N_2_ at 77 K and CO_2_ at 195 K after evacuation for 24 h under vacuum at 60 °C. No uptake was observed for N_2,_ but a two-step CO_2_ isotherm was observed with a type F–I isotherm profile (Figure 5c). An initial steep uptake of ca. 1.56 mmol/g of CO_2_ at 13.0 mbar preceded a linear uptake profile, indicating a gradual phase transition that plateaued with uptake of 3.66 mmol/g of CO_2_ at 1 atm. A similar stepped isotherm was observed for C_2_H_2_ adsorption (Figures 5d and S22) wherein ca. 1.32 mmol/g of C_2_H_2_ was adsorbed up to 332 mbar at 298 K, followed by a gradual transition to a new phase with 2.28 mmol/g uptake at 1 atm. Type I gas sorption isotherm profiles were recorded for C_2_H_4_ and C_2_H_6_ with uptakes of 0.89 and 0.52 mmol/g, respectively. Gas sorption experiments were also performed for C3 hydrocarbons, which all displayed type I isotherms with C_3_H_4_ uptake of 1.90 and 1.64 mmol/g at 273 and 298 K, respectively, as well as 1.04 and 0.49 mmol/g for C_3_H_6_ and C_3_H_8_, respectively, at 298 K (Figure 5e). All gas sorption experiments were replicated on **2** synthesized by slurry (Figure S21c–f) which afforded similar results, although typically with lower uptake and larger hysteresis for some experiments (e.g., CO_2_ 195 K and C_2_H_2_ 298 K). In the case of C_2_H_2_ adsorption, the gate-opening shifted from 360 mbar for the layering-based product to 670 mbar for the slurry-based product, a feature also seen in the water sorption isotherms. This phenomenon has been observed previously for DUT-8 and other structurally flexible compounds by Kaskel et al.^41,42^

The selectivity of **2** for C_2_H_2_ suggested the possibility of separating C_2_H_2_ from industrially relevant gas mixtures. Whereas ideal absorbed solution theory (IAST)^43−45^ calculations can serve as a good indicator for separation performance for single-gas 298 K adsorption isotherms, care must be taken in the case of flexible materials, and so IAST calculations were performed using C_2_H_2_ isotherm values prior to the phase transformation (Table S7). Calculated selectivity values for 1:1, 1:9, and 1:99 C_2_H_2_:C_2_H_4_ were 15.18, 25.81, and 33.01, and for 1:1, 1:9, and 1:99 C_2_H_2_:CO_2_ were 10.90, 14.75, and 16.94, respectively, at 1 bar, 298 K (Figure 5f). The 1:1, 1 bar 298 K C_2_H_2_:CO_2_ selectivity exceeds that of the sulfate-pillared material SOFOUR-1-Zn (6.6), although it underperforms vs the current benchmark sorbent, SOFOUR-TEPE-Zn (16833). Among materials with reported 1:99 C_2_H_2_:C_2_H_4_ selectivity, **2** lies between TIFSIX-2-Ni (22.7)^46^ and ZJU-74a (24.2)^47^ and stronger performing sorbents such as NKMOF-1-Ni (44.0),^48^ ZJU-280a (44.5),^49^ TIFSIX-2-Cu-i (55),^50^ ZUL-100/200/300 (175/114/139),^51,52^ MFSIX-14-Cu-i (M = Si, Ge, Ti for UTSA-200a (6320),^29^ ZU-33 (>1100),^53^ and ZU-13 (229),^54^ respectively), Co(4-DPDS)_2_CrO_4_ (834),^55^ and NCU-100a (7291)^28^ (Figure S30, Table S8).

The separation performance of **2** was experimentally determined by fixed-bed dynamic column breakthrough experiments using 1.40 g of a slurry-synthesized batch of **2np** and 1:1, 1:9, and 1:99 C_2_H_2_:CO_2_ or C_2_H_2_:C_2_H_4_ at RT, 1 bar, with a combined flow rate of 1 sccm for 1:1, 2 sccm for 1:9, or 5 sccm for 1:99 gas mixtures. **2np** separated C_2_H_2_:CO_2_ with breakthrough times of ca. 14.3, 42.9, and 60.7 min/g for 1:1, 1:9, and 1:99 C_2_H_2_:CO_2_, respectively (Figures S24–S26), C_2_H_2_:C_2_H_4_ was separated with breakthrough times of ca. 17.9, 32.1, and 39.3 min/g for 1:1, 1:9, and 1:99, respectively (Figures S27–S29). We attribute this separation performance to the narrow-pore structure of **2np** and the C_2_H_2_ interactions with sulfate anions. The disordered structure of **2np** (Tables S4 and S5) impedes the determination of more detailed gas binding sites through computational modeling.

## 3 Conclusions

To conclude, we report that structural flexibility can exist in PCNs with a **sql** network topology formed with a rare CuSO_4_ MBB dimer. The use of 1,4-bib as a linker ligand resulted in a known structure, **1**, that transformed between cp and op structures during H_2_O or CO_2_ sorption. When the bulkier 1,4-bin ligand was used to form **2**, flexibility between op and np phases was observed, resulting in stepped isotherms for H_2_O and C_2_H_2_, and C_2_H_2_ selectivity for C_2_H_2_:CO_2_ or C_2_H_2_:C_2_H_4_ gas mixtures. **1** and **2** exemplify the two structural variants of this rare CuSO_4_ MBB, the “Structure A” variant in **1** and the “Structure B” variant in **2op**, both enabling flexibility but with different mechanisms and sorption properties. It is unclear whether Structures A and B are responsible for the observed differences in structure–property relationships. Further exploration of the CuSO_4_ MBB and the effect of ligand bulkiness and/or torsional flexibility is needed in order to address these differences.

## Supporting Information Available

Experimental section; Cambridge structural database (CSD) analysis of CuSO_4_ MBB dimer; CuSO_4_(1,4-bib)1.5, **1**; and CuSO_4_(1,4-bin)1.5, **2** (PDF)

## Abbreviations

PCN: porous coordination network
MBB: molecular building block
SCXRD: single-crystal X-ray diffraction
PXRD: powder X-ray diffraction
VTPXRD: variable-temperature powder X-ray diffraction
TGA: thermogravimetric analysis
DSC: differential scanning calorimetry
DVS: dynamic vapor sorption
IAST: ideal adsorbed solution theory
