# Supplementary material for: Dinuclear Copper Sulfate-Based Square Lattice Topology Network with High Alkyne Selectivity
Source: Cryst Growth Des. 2024 Feb 27;24(6):2573–9. doi: 10.1021/acs.cgd.4c00094 (PMC10958442; doi:10.1021/acs.cgd.4c00094)
Supplement: Supplementary file 1 — cg4c00094_si_001.pdf [file cg4c00094_si_001.pdf]

Supplementary Information

Dinuclear copper sulfate-based square lattice topology network with high alkyne selectivity

*Yassin H. Andaloussi, Debobroto Sensharma, Andrey A. Bezrukov, Dominic C. Castell, Tao He, Shaza Darwish, and Michael J. Zaworotko**

Department of Chemical Sciences and Bernal Institute, University of Limerick, Co. Limerick, Y94 T9PX, Ireland.

*Email: Michael.Zaworotko@ul.ie

Contents

1 Experimental section 4
- 1.1 Materials and synthesis 4
  - Synthesis of 1,4-bis(1-imidazolyl)benzene (1,4-bib) 4
  - Synthesis of $CuSO_4$(1,4-bib)$_{1.5}$, **1**, bulk powder 4
  - Synthesis of $Cu_2(SO_4)_2$(pyridine)$_6$ 4
  - Synthesis of $CuSO_4$(1,4-bib)$_{1.5}$, **1op**, single crystals 5
  - Synthesis of closed phase of $CuSO_4$(1,4-bib)$_{1.5}$, **1cp** 5
  - Synthesis of 1,4-bis(1-imidazolyl)naphthalene (1,4-bin) 5
  - Synthesis of $CuSO_4$(1,4-bin)$_{1.5}$, **2**, bulk powder 5
  - Synthesis of $CuSO_4$(1,4-bin)$_{1.5}$, **2op**, single crystals 6
  - Synthesis of narrow pore phase of $CuSO_4$(1,4-bin)$_{1.5}$, **2np** 6
- 1.2 Powder X-ray diffraction measurements 6
  - Powder X-Ray Diffraction (VT-PXRD) under ambient conditions 6
  - In-situ Variable Temperature Powder X-Ray Diffraction (VT-PXRD) 6
- 1.3 Single-Crystal X-ray Data Collection and Structure Determination 7
  - Specific Refinement Details – **1** 8
  - Specific Refinement Details – **2** 8
- 1.4 Thermogravimetric analysis (TGA) and Differential Scanning Calorimetry (DSC) 10
- 1.5 Sorption 10
  - Low-pressure gas sorption measurements 10
  - Low pressure gas adsorption of a room temperature-activated sample of $CuSO_4$(1,4-bib)$_{1.5}$, **1op** 11
  - High-pressure CO_2 adsorption measurements 11
  - Water Vapor Sorption 11
  - Dynamic column Breakthrough (DCB) 12

2 Cambridge structural database (CSD) analysis of $CuSO_4$ MBB dimer 12

3 $CuSO_4$(1,4-bib)$_{1.5}$, 1 14
- 3.1 Crystal structure of **1** 14
- 3.2 Thermogravimetric analysis (TGA) of **1op** 17
- 3.3 Differential scanning calorimetry (DSC) for **1op** 17
- 3.4 Crystallographic information for **1** 18
- 3.5 Overlay of **1op** at 100 K and **1cp** at 343 K 19
- 3.6 Variable Temperature Powder X-ray diffraction (VTPXRD) of **1** 20
- 3.7 Dynamic vapor sorption (DVS) of **1** 21
- 3.8 High Pressure CO_2 sorption of **1** 22

3.9 Low pressure CO_2, C_2H_2, C_2H_4, C_2H_6 sorption at 298 K of **1** 23
3.10 RT-activated 298 K CO_2 sorption of **1op** 23
3.11 Slurry-based synthesis of **1** 24
3.12 PXRD after thermal decomposition of **1** 24
4 $CuSO_4(1,4\text{-bin})_{1.5}$, **2** 25
4.1 Crystal structure of **2** 25
4.1 Overlay of **2op** and **2np** 29
4.2 Thermogravimetric analysis (TGA) of **2op** 30
4.3 Differential scanning calorimetry (DSC) for **2op** 30
4.4 Variable Temperature Powder X-ray diffraction (VTPXRD) of **2** 31
4.5 PXRD, sorption and IAST selectivity for **2**-Slurrying 32
4.6 PXRD after thermal decomposition of **2** 33
4.7 IAST Selectivity Calculations 33
4.8 Dynamic Column Breakthrough (DCB) experiments 34
4.9 Gas sorption comparison with leading materials 37
5 References 39

1 Experimental section

1.1 Materials and synthesis

1,4-bis(imidazol-1-yl)benzene (1,4-bib) and $Cu_2(SO_4)_2$(Pyridine)$_6$ was synthesized using modified reported procedures.[1, 2] Other reagents and solvents were commercially available and were used without further purification.

Synthesis of 1,4-bis(1-imidazolyl)benzene (1,4-bib)

1,4-dibromobenzene (5.0 g, 21.2 mmol, 1.0 eq), CuI (805 mg, 20 mol%), K_2CO_3 (8.78 g, 63.6 mmol, 3.0 eq) and imidazole (4.33 g, 63.6 mmol, 3.0 eq) were added to anhydrous DMF (50 mL) under N_2. The reaction was then stirred and heated to 150 °C for 48 h under N_2. After cooling to room temperature, the mixture was diluted with DCM (250 mL) and filtered. The filtered organic layer was transferred to a large separating funnel and washed with H_2O (2 × 500 mL). After drying over $MgSO_4$, the organic layer was concentrated under reduced pressure. Final purification was achieved by rapid trituration of the compound from a DCM/hexane mixture, affording 1,4-bib as a white solid (4.10 g, 92%). ^{1}H NMR (400 MHz, DMSO-*d$_6$*) δ 7.11 (br, s, 2H), 7.71-7.88 (m, 6H), 8.32 (br, s, 2H).

Synthesis of $CuSO_4$(1,4-bib)$_{1.5}$, 1, bulk powder

Layering: In a test tube, a solution of $CuSO_4 \cdot 5H_2O$ (25.0 mg, 0.1 mmol, 1 eq) is dissolved in 2 mL H_2O. Above this, a buffer layer of 4 mL 1:1 (v/v) MeOH:H_2O solution is slowly added. A final layer is then slowly added of 1,4-bis(1-imidazolyl)benzene (21.0 mg, 0.1 mmol, 1 eq) in 2 mL MeOH and left to stand for *ca*. 3 weeks. The product is extracted as a dark blue powder that deposits as a layer towards the middle of the test tube which is extracted, filtered under reduced pressure, and washed with H_2O. Yield: 18.0 mg, 52% yield based on $CuSO_4$(1,4-bib)$_{1.5}\cdot$2.448(H_2O). **1** samples made through this layering method were, unless otherwise stated, used for all further experiments.

Slurrying: Alternatively, a solution of $CuSO_4 \cdot 5H_2O$ (16.7 mg, 0.067 mmol, 0.66 eq) in 2 mL H_2O is added dropwise to a stirring solution of 1,4-bis(1-imidazolyl)benzene (21.0 mg, 0.1 mmol, 1 eq) in 2 mL MeOH and left for 24 h. The resultant product is filtered and washed with 2 mL H_2O to yield a dark blue powder. Yield: 15.8 mg, 45% yield based on $CuSO_4$(1,4-bib)$_{1.5}\cdot$2.448(H_2O).

Synthesis of $Cu_2(SO_4)_2$(pyridine)$_6$

$CuSO_4 \cdot 5H_2O$ (300 mg, 1.2 mmol) is stirred and heated in a capped vial to 80 °C for 24 h in 10 mL Pyridine. The solution is then allowed to cool to room temperature, then filtered under reduced pressure. Characterization data from PXRD match with a simulated PXRD of the reported material (Refcode: CEVJUL).[2] Yield: 298.6 mg, 48% yield.

Synthesis of $CuSO_4$(1,4-bib)$_{1.5}$, 1op, single crystals

In a test tube, a solution of $Cu_2(SO_4)_2$(pyridine)$_6$ (40.1 mg, 0.05 mmol, 0.5 eq) is dissolved in 2 mL H_2O. Above this, a layer of 4 mL 1:1 (v/v) MeOH:H_2O solution is slowly added. This is carefully layered with a solution of 1,4-bis(1-imidazolyl)benzene (21.0 mg, 0.1 mmol, 1 eq) in 2 mL MeOH and left to stand for ca. 1 week. From this, large rectangular dark blue block-shaped crystals of **1op** that were suitable for single-crystal X-ray diffraction analysis are collected.

Synthesis of closed phase of $CuSO_4$(1,4-bib)$_{1.5}$, 1cp

The closed phase, **1cp**, was formed by heating the sample to 100 °C under high vacuum or under N_2. However, given the water vapor uptake at low relative humidity the sample must be maintained under a dry atmosphere to maintain the closed phase. Single crystals of **1cp** could be formed from **1op** by heating the crystal to 70 °C for 1h under a flow of N_2.

Synthesis of 1,4-bis(1-imidazolyl)naphthalene (1,4-bin)

A mixture of 1,4-dibromonaphthalene (10.0 g, 35.0 mmol, 1 eq), imidazole (14.3 g, 209.8 mmol, 8.3 eq), K_2CO_3 (14.5 g, 104.9 mmol, 3 eq), and $CuSO_4$ (111.6 mg, 0.7 mmol, 0.02 eq) were stirred at 180 °C for 24 h in a round-bottom flask (100 mL) under N_2 atmosphere. After cooling down to room temperature, the crude product was washed with H_2O (50 mL × 3). The solid residue was then dissolved in MeOH then filtered then the resultant filtrate was concentrated to give 1,4-di(1H-imidazol-1-yl)naphthalene as a light yellow solid (7.3 g, yield: 80.2%). ^{1}H NMR (400 MHz, DMSO-d_6) δ 7.22 (s, 2H), 7.56-7.58 (m, 2H), 7.61 (s, 2H), 7.69-7.71 (m, 4H), 8.06 (s, 2H).

Synthesis of $CuSO_4$(1,4-bin)$_{1.5}$, 2, bulk powder

Layering: In a test tube, a solution of $CuSO_4{\cdot}5H_2O$ (25.0 mg, 0.1 mmol, 1 eq) is dissolved in 2 mL H_2O. Above this, a buffer layer of 4 mL 1:1 (v/v) MeOH:Ethylene glycol solution is slowly added. A final layer is then slowly added of 1,4-bis(1-imidazolyl)naphthalene (26.0 mg, 0.1 mmol, 1 eq) in 2 mL MeOH and left to stand for *ca*. 1 month. The product is extracted as a dark blue powder that deposits as a layer towards the middle of the test tube which is extracted, filtered under reduced pressure, and washed with MeOH. Solvent exchange with MeOH is then performed by adding 1 mL MeOH before decanting and replacing with fresh MeOH three times per day over the course of 3 days. Yield: 15.7 mg activated, 43% yield. **2** samples made through this layering method were, unless otherwise stated, used for all further experiments.

Slurrying: Alternatively, $CuSO_4{\cdot}5H_2O$ (1.25 g, 5.0 mmol, 0.66 eq) and 1,4-bis(1-imidazolyl)naphthalene (1.955 g, 7.5 mmol, 1 eq) is added to 250 mL MeOH and stirred for 4 days. The resultant product is filtered and washed with 20 mL MeOH then left to dry in air yielding a light blue powder. Yield: 2.576 g activated, 94% yield.

Synthesis of $CuSO_4$(1,4-bin)$_{1.5}$, 2op, single crystals

In a test tube, a solution of $Cu_2(SO_4)_2$(pyridine)$_6$ (20.0 mg, 0.025 mmol, 1 eq) is dissolved in 2 mL distilled water H_2O. Above this, a layer of 4 mL 1:1 (v/v) MeOH:H_2O solution is slowly added. This is carefully layered with a solution of 1,4-bis(1-imidazolyl)naphthalene (6.5 mg, 0.025 mmol, 1 eq) in 2 mL MeOH and left to stand for ca. 1 week. From this, large dark blue wedge-shaped crystals that were suitable for single-crystal X-ray diffraction analysis are collected.

Synthesis of narrow pore phase of $CuSO_4$(1,4-bin)$_{1.5}$, 2np

The closed phase was formed by heating **2op** to 60 °C under atmospheric conditions, or exposure to low humidity (<20% RH) air, high vacuum or under N_2. Single crystals of **2np** could be formed from **2op** by placing the crystal at *ca.* 275 K for 1h under a flow of N_2 (or in one instance at 250 K for 1h under a flow of N_2), however careful control is needed to prevent the single crystal from cracking extensively during the phase change.

1.2 Powder X-ray diffraction measurements

Powder X-Ray Diffraction (PXRD) under ambient conditions

PXRD experiments were conducted using microcrystalline samples on a PANalytical Empyrean diffractometer (40 kV; 40 mA; Cu $K\alpha_{1,2}$ λ = 1.5418 Å) in Bragg–Brentano geometry. The sample was exposed for 99.5 s/step, with a step size of 0.0131303° in 2θ, at room temperature with a range of 2°-50°. Powder samples were evenly distributed on a zero-background holder after being lightly ground to minimize the effects of preferred orientation. Data analysis was carried out using X'Pert HighScore Plus[3] (version 2.2e). Powder patterns were calculated from SCXRD structures using Mercury (v.2023.1.0).[4]

In-situ Variable Temperature Powder X-Ray Diffraction (VTPXRD)

Diffractograms at different temperature were recorded using a PANalytical X'Pert Pro-MPD diffractometer equipped with a PIXcel3D detector operating in scanning line detector mode with an active length of 4 utilizing 255 channels. An Anton Paar TTK 450 stage coupled with the Anton Paar TCU 110 Temperature Control Unit was used to record the variable temperature diffractograms. The diffractometer is outfitted with an Empyrean Cu LFF (long fine-focus) HR (9430 033 7300x) tube operated at 60 kV and 60 mA and Cu Kα radiation (λα = 1.5418 Å) was used for diffraction experiments. Continuous scanning mode with the goniometer in the theta-theta orientation was used to collect the data. Incident beam optics included the Fixed Divergences slit, with a 1/4° divergence slit and a Soller slit (0.04 rad). Divergent beam optics included a P7.5 S7 anti-scatter slit, a Soller slit (0.04 rad), and a Ni-β filter. *Ca*. 20 mg of freshly synthesized **1** or **2** was ground into a fine powder, and then loaded onto a zero-background sample holder made for an Anton Paar TTK 450 chamber. The data was collected from 4°-40° (2θ) with a step-size of 0.0167113° and a scan time of 200 seconds per step. Crude data were analyzed using the X'Pert HighScore Plus™ software V 4.1 (PANalytical, The Netherlands). PXRD was performed first in ambient air, then in dry N_2 flow before the sample was heated, up to 473 K for **1** or up to 393 K for **2**. The samples were equilibrated for 10 min at each new temperature step (or after N_2 flow begins) before data collection. The samples were then cooled to

room temperature, still under N_2 flow, then measurements were performed, then the TTK 450 stage was opened to allow the sample to be exposed to ambient air for 10 min, followed by a final measurement.

1.3 Single-Crystal X-ray Data Collection and Structure Determination

For **1**, two suitably large plate crystals (dimensions: Crystal 1: 0.38 x 0.295 x 0.075 mm or Crystal 2: 0.19 x 0.168 x 0.02 mm) were selected for single-crystal analysis. Using a nitrogen-flow Oxford cryostream attachment, the first crystal was cooled to 100 K at a rate of 360 K/h from room temperature. A full data set of **1op** was collected at this temperature. The crystal was then heated to room temperature (~299 K) at a rate of 360 K/h and a full data set was collected. The crystal was then heated to 333 K at a rate of 60 K/h and then held at this temperature for 1 h. A full data set was then collected at this temperature. A second crystal was heated from room temperature to 343 K at 60 K/h and held at this temperature for 1 h to form **1cp**. A short data collection at this point resulted in a diffraction pattern with sharply reduced diffraction intensity and a smaller unit cell indicating the phase change. A full data collection was repeated at this temperature from which the closed-phase crystal structure was determined.

For **2**, a suitably large crystal (0.3 × 0.15 × 0.05 mm) was selected for single crystal refinement. Using a nitrogen-flow Oxford cryostream attachment, the crystal was cooled to 100 K at a rate of 360 K/h from room temperature. A full data set of **2op** was collected at this temperature. A second large crystal (0.42 × 0.324 × 0.194 mm) was heated from 100 K to 250 K at a rate of 360 K/h, and then steadily raised to 276 K at a rate of 30 K/h until signs of phase transition to **2np** became visible (discoloration and cracking). Note, in the vast majority of cases, this phase transition resulted in crystals too disfigured for SCXRD. The crystal was held at this temperature for 30 min, then heated to 333 K at a rate of 240 K/h, then to 373 K at a rate of 240 K/h to ensure full desolvation, then cooled to 100 K at a rate of 360 K/h. Data collection was performed at this temperature from which the activated crystal structure was determined. A third large crystal of **2op** (0.69 x 0.25 x 0.17 mm) was subjected to data collection at room temperature. To prevent conversion to **2np** through exposure to dry air, the nitrogen flow Oxford cryostream attachment was turned off, thus inhibiting thermal control. A fourth large crystal of **2op** (0.306 x 0.27 x 0.15 mm) was placed on the preheated diffractometer for 1 h at 250 K using the nitrogen flow Oxford cryostream attachment. Minor cracking and discoloration indicated conversion to **2np**, which was confirmed through unit cell determination. The crystal was heated to 298 K at a rate of 60 K/h and a data set was collected.

Data collection was performed with a Mo Kα (λ = 0.71073 Å) radiation source on a Bruker D8 Quest fixed-chi diffractometer equipped with a Bruker APEX-II CCD detector. Data was indexed, integrated, and scaled in APEX4.[5] Absorption corrections were performed by the multi-scan method using SADABS[6]. Space groups were determined using XPREP[7] as implemented in APEX4. The SHELX-2014 program package, implemented in OLEX2 v1.5, [8] was used for structure solution and refinement. Structures were solved using the intrinsic phasing method (SHELXT)[9] and refined with SHELXL[10] using the least-squares method. All non-hydrogen atoms were refined anisotropically. Hydrogen atoms were placed in calculated positions from the molecular geometry and assigned isotropic thermal parameters that depended on the equivalent displacement parameters of their carriers. Selected crystallographic data and refinement parameters for the crystal structures are given in Tables S3 and S5.

Specific Refinement Details – 1

As **1** is heated from 100 K to 333 K, the open phase is retained, however water molecules in the pore (O5, O6, and O7) occur with progressively lower occupancy or are no longer present entirely.

For **1op** at RT, this results in a low occupancy for O6 which can only be modelled isotropically, without bonded hydrogen, and with large U_{iso} value. As such the position of this atom is inaccurate resulting in an unusually close O3-O6 contact.

For **1op** at 333 K, when the OLEX2 implementation of SQUEEZE[11] is used to account for the remaining electron density in the pore only 4 electrons were found, which could only account for *ca*. 20% of a single water molecule per Asymmetric Unit. A sufficient model was therefore chosen without SQUEEZE wherein the pore was modelled as empty, which is supported by Dynamic Vapor Sorption (DVS) experiments which show the weakly-bound water is removed at 298 K under dry air, and so is conceivably not present in the single crystal at 333 K under a dry N_2 flow.

1op at RT

Alert level B

```
PLAT430_ALERT_2_B Short Inter D...A Contact O3 ..O6 . 2.64 Ang. x,y,z = 1_555 Check
```

Response: Occupancy of O6 is low (0.26) and so position is inaccurate making the O3-O6 distance unreliable.

1op at 333 K

Alert level B

```
PLAT601_ALERT_2_B Unit Cell Contains Solvent Accessible VOIDS of 117 Ang**3
```

Response: As the structure is collected at 333 K, this void space is expected to be empty as is confirmed by thermogravimetric analysis.

Specific Refinement Details – 2

For **2**, both **2op** and **2np** displayed disorder in the naphthalene and sulfate region, with **2np** also showing disorder in the imidazole region of the structure.

For **2op** at 100 K, disorder in the naphthalene arises from the naphthalene of the single-ligand wall crystallizing onto a center of inversion. Additionally, solvent water molecules could not be adequately resolved and so were largely accounted for using the OLEX2 implementation of the SQUEEZE[11] routine, which found 46 electrons in the pore which approximately accounts for 4.33 water molecules. Water molecules based on O5 and O6 could not be handled with SQUEEZE due to being co-located with disordered regions of naphthalene, and so were each individually resolved with a fixed 0.33 occupancy. Disorder in the sulfate group is evident from the presence of Q-peaks with intensities of 1.6, 1.3, and 1.0 adjacent to sulfate atoms. Splitting the sulfate group into the new positions with 14.5% freely refined occupancy, as indicated by the Q-peaks, resulted in the R_1 dropping from 4.21% to 3.40%. Disorder of this kind in the sulfate group is potentially supported by the existence of two known structural variants of the copper sulfate dimer MBB in the CSD wherein the Cu-O2 distance (for more information see section 2) varies between ~2.6-2.9 Å and ~3.0-3.3 Å. In this case, the major and minor components have Cu-O2 distances of 2.7607(17) Å and 3.170(15) Å, respectively, and so would appear

to meet this trend. However, it should be noted that due to the low occupancy of the minor component, the measured distances are less reliable than would be indicated by estimated standard deviation.

For **2op** at RT, similar disorder was observed in the naphthalene, sulfate, and O5/O6 water molecules of the structure. Additional pore solvent water molecules were accounted for with SQUEEZE which found 48 electrons in the pore approximately accounting for 4.33 water molecules. Disorder in the sulfate region was resolved with 14.4 % freely refined occupancy.

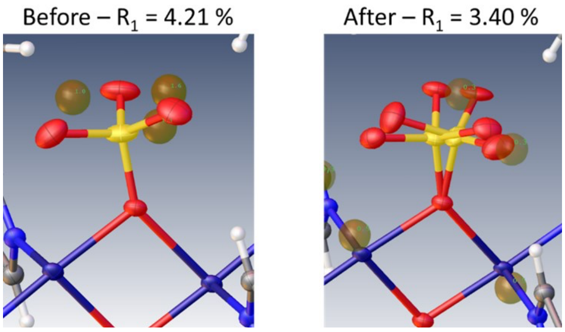

Figure S1: Q-peaks and crystallographic disorder in **2op** at 100 K before and after disorder is resolved, resulting in a reduction in R_1.

For **2np** at 100 K, cracking of the crystal upon in-situ desolvation by exposure to dry nitrogen in the cryostream results in severely reduced data quality. Attempts to form the activated phase crystals through other means, such as by gradually reducing relative humidity in a dynamic vapor sorption instrument, or exposure to reduced atmospheric pressure, or by heating, failed to yield suitable crystals. As with the as-synthesized phase, the structure presents with disorder about the naphthalene group of the single-ligand wall which crystallizes onto a center of inversion. Additionally, Q-peaks surrounding the imidazole group of the single-wall ligand and the sulfate indicate the presence of disorder in these groups, with the imidazole group rotated 88.46(17)° between disordered forms, and the sulfate group changing from a monoatomic bridging group (μ_2-η^2) to a diatomic bridging group (μ_2-η^1:η^1). Both minor components are refined with a fixed 25% occupancy. Due to poor data quality, and the extensive disorder, the structure is heavily restrained (SADI, RIGU, and ISOR) and constrained (EADP) to produce a stable refinement, and so all measurements taken from this structure should be treated with extreme caution.

For **2np** at 298 K, cracking of the crystal upon in-situ desolvation reduced data quality. Similar disorder presents at 298 K as at 100 K, with the imidazole group rotated 88.8(8)° between disordered forms and sulfate group disorder between monoatomic and diatomic bridging refined with a fixed 25% occupancy. Due to poor data quality, the structure is restrained (SADI, RIGU), and so all measurements taken from this structure should be treated with significant caution.

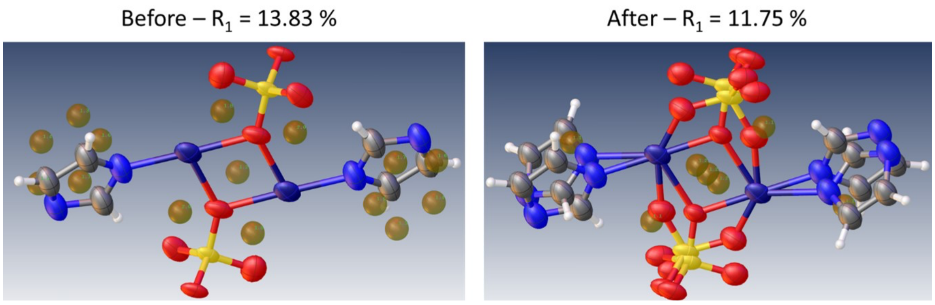

Figure S2: Q-peaks and crystallographic disorder in **2np** at 100 K before and after disorder is resolved, resulting in a reduction in R_1.

2np at 100 K

Alert level B

```
PLAT341_ALERT_3_B Low Bond Precision on C-C Bonds .............. 0.01777 Ang.
```

Response: Crystal heavily cracked due to phase change and significant disorder is present and so data quality is poor.

Alert level C

```
PLAT029_ALERT_3_C _diffrn_measured_fraction_theta_full value Low . 0.974 Why?
```

Response: The cryojet is blocking a portion of the detector so calculated strategy does not find all peaks.

```
PLAT082_ALERT_2_C High R1 Value .................................. 0.12 Report
```

Response: Crystal heavily cracked due to phase change and significant disorder is present and so data quality is poor.

```
PLAT084_ALERT_3_C High wR2 Value (i.e. > 0.25) .................. 0.33 Report
```

Response: Crystal heavily cracked due to phase change and significant disorder is present and so data quality is poor.

```
PLAT601_ALERT_2_C Unit Cell Contains Solvent Accessible VOIDS of . 52 Ang**3
```

Response: As the crystal is heated to 100 °C the pore is expected to be empty. The SQUEEZE routine was not found to improve data quality.

1.4 Thermogravimetric analysis (TGA) and Differential Scanning Calorimetry (DSC)

Thermogravimetric analysis was performed under N_2 flow using a TA Instruments Q50 system. A sample was loaded into an aluminum sample pan and heated at 10 °C min^{-1} from room temperature to 400 °C. Differential scanning calorimetry was carried out using a TA Instruments Q2000 differential scanning calorimeter. The sample and reference pans were heated at 10 °C min^{-1} from room temperature to 400 °C and so the heat flow, relative to the reference, was measured as a function of time and temperature under a controlled atmosphere. N_2 gas flowing at a rate of 50 mL min^{-1} was used to purge the furnace.

1.5 Sorption

Gases were used as obtained from BOC Gases (Ireland), with the following certified purities: He (99.999%), CO_2 (99.995%), C_2H_2 (98.5%), C_2H_4 (99.92%), C_2H_6 (99.0%), C_3H_4 (97.0%), C_3H_6 (99.5%), C_3H_8 (99.95%), N_2 (99.998%).

Low-pressure gas sorption measurements

The sorption isotherms for N_2 at 77 K, and CO_2 or measured using a Micromeritics 3 Flex surface area and pore size analyzer. Bath temperatures of 77 K and 195 K were maintained using liquid nitrogen

and a dry ice–acetone slurry, respectively. Before gas sorption experiments, the freshly prepared samples of **1** or **2** were placed in the quartz tube and degassed under high vacuum at 100 °C or 60 °C, respectively, for 24 h in a Micromeritics SmartVacPrep system to remove the remnant solvent molecules prior to measurements. For sorption isotherms of CO_2, C_2H_2, C_2H_4, C_2H_6, C_3H_4, C_3H_6, C_3H_8 at 273 K, and 298 K, a Julabo temperature controller containing an ethylene glycol/water mixture was used to maintain a constant temperature in the bath through the duration of the experiment.

Low pressure gas adsorption of a room temperature-activated sample of $CuSO_4(1,4\text{-bib})_{1.5}$, 1op

A fresh, water-loaded, sample of $CuSO_4(1,4\text{-bib})_{1.5}$, **1op**, was evacuated directly within the micromeritics 3 Flex surface area and pore size analyser at room temperature for 1 h (to allow loosely bound water to desorb, while retaining strongly bound water and consequently the **1op** phase) prior to data collection of a CO_2 isotherm at 195 K. Two subsequent cycles of CO_2 195 K isotherms were performed directly on the sample without reactivation, each with 1 h evacuation within the micromeritics 3 Flex surface area and pore size analyser. A final measurement was then performed after the sample was activated under high vacuum in a Micromeritics SmartVacPrep system at RT for 24 h.

High-pressure CO_2 adsorption measurements

High-pressure CO_2 sorption measurements were performed using a Hiden Isochema XEMIS microbalance. A sample of **1** that was activated on a Micromeritics SmartVacPrep system at 100 °C for 24 h was transferred to the XEMIS system and then further outgassed under secondary vacuum for 3 h *in situ* before isotherms were collected. Excess adsorption and desorption profiles were obtained after applying buoyancy correction using the crystallographically determined skeletal density of the **1op** at 100 K. Temperatures were maintained at 273 K, 298 K, 303 K, 308 K, or 313 K using a Grant LT Ecocool 150 temperature controller.

Water Vapor Sorption

Water vapor sorption was performed using an Adventure dynamic vapor sorption (DVS) instrument manufactured by Surface Measurement Systems. The instrument measures water vapor uptake gravimetrically using air as a carrier gas. A water vapor-saturated flow is created by passing dry air through a water bubbler. Specified relative humidity is generated through precise mixing of dry and saturated gas flows in desired, calibrated flow ratios. Digital mass flow controllers regulate flows of dry and saturated gases. Pure water was used to generate water vapor for these measurements and the temperature was maintained at the desired level (either 298 K, 300 K or 333 K) by enclosing the system in a temperature-controlled incubator. The mass of the sample was determined by the high-resolution microbalance Ultrabalance Low Mass with a precision of 0.01 µg. The microbalance has a symmetric configuration with both the sample pan and reference pan being exposed to the same gas and being kept at the same temperature, allowing negation of buoyancy and drag effects. The instrument is equipped with two such balances, allowing measurement of the two samples in parallel. Prior to the measurement, each sample was activated in-situ in dry air at 60-100 °C for 60 minutes using the built-in preheater and consequently cooled to sorption temperature over 90 minutes. Isotherm measurements were performed on approximately 10 mg of sample powder. For each isotherm point, $dm/dt < 0.05\%\ min^{-1}$ for a minimum of 10 minutes was used as criteria of reaching equilibrium.

Dynamic column Breakthrough (DCB)

Dynamic breakthrough experiments were performed on a custom-made rig with **2**, as synthesized by slurrying. In a typical experiment, ~1.4 g of the pre-activated **2np** sample was packed into quartz tubing (8 mm diameter) to form a fixed bed. The sample was activated before each experiment by purging with 20 cm^3 min^{-1} of He gas at 333 K for 12 h. After cooling to room temperature, the gas flow was switched to the desired composition of C_2H_2/C_2H_4 or C_2H_2/CO_2 which was pre-equilibrated through a separate column, and regulated by mass flow controllers. The outlet composition was monitored at 5 min intervals by gas chromatography (Shimadzu, GC 2030, Flame Ionization Detector (FID) for C_2H_2 and C_2H_4 and Thermal Conductivity Detector (TCD) for CO_2).

2 Cambridge structural database (CSD) analysis of $CuSO_4$ MBB dimer

A CSD survey was conducted using ConQuest (2023.2.0) and the results were processed with Mercury (v.2023.2.0) to find all instances of the $CuSO_4$ MBB dimer using the following search query:

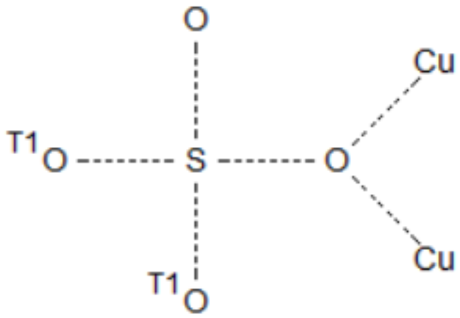

Two oxygen atoms were limited to being one-connected (T1) to reduce the number of unrelated hits. No further restrictions were added. This results in 48 total hits from which 24 hits contain the $CuSO_4$ MBB dimer which are listed in Table S1.

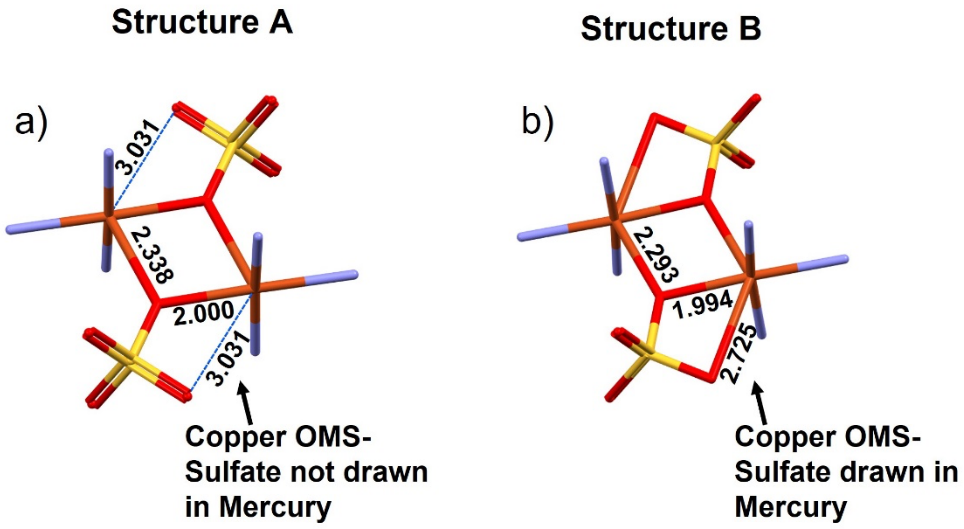

Figure S3: Comparison of known structural variations of the $CuSO_4$ MBB dimer in (a) CSD refcode: PIGYEK and (b) CSD refcode: CEVJUL showing that the interaction between SO_4 and the Cu open metal site (OMS) is in some cases within or beyond the standard Mercury Cu-O bond distance cut-off of 2.80 Å.

Table S1: List of known structures in the CSD featuring the $CuSO_4$ MBB dimer.

Refcode	Dimensionality	Void space (When solvent removed)	Structure A/B in CSD?	Sulfate-Cu OMS bond length (Å)?	DOI
APIQIA	0D	0.9%	A	3.209	10.1134/S0036023610090093[12]
CEVJUL	0D	0%	B	2.725	10.1107/S2053229618001547[2]
ERORAE	0D	9.5%	A	2.688	10.1002/ejic.200300426[13]
FENMIU	0D	1.9%	A	3.128	10.1039/dt9870000493[14]
FENMIU01	0D	2.9%	A	3.117	CSD Communication (2015)
HULTIT	1D/2D*	0%	A	2.846	CSD Communication (2015)
HULTIT01*	N/A	N/A	N/A	N/A	10.1016/j.poly.2016.03.025[15]
LALFIO	2D	2.9%	A	2.872	10.1039/b419418g[16]
MUSLUI	0D	8.2%	A	2.738	10.1016/j.ica.2010.01.006[17]
MUZQIK	0D	9.7%	A	3.131	10.1016/j.molliq.2020.112590[18]
NUCCAR	2D	47.5%	A	3.229	10.1039/C5DT00011D[19]
OVADUL	0D	14.4%	B	2.709	10.1016/j.inoche.2011.02.028[20]
PIGYEK	2D	9.0%	A	3.031	10.5517/ccpts8j [21]
POQCEF	0D	0%	A	3.103	CSD Communication (2014)
PUNGAJ	0D	11.3%	A	3.141	10.1016/j.poly.2020.114624[22]
QICZIP	0D	6.4%	A	3.046	10.1002/slct.201700917[23]
RIPJUW	0D	13.1%	A	2.888	10.1039/b708465j[24]
ROFMUX	0D	0%	B	2.793	10.1107/S2053229619004625[25]
TAGROK	0D	21.9%	B	2.701	10.1016/j.ica.2010.08.018[26]
TAGSAX	0D	9.1%	B	2.769	10.1016/j.ica.2010.08.018[26]
TOSSEB	0D	0%	A	3.454	10.1039/C4DT03393K[27]
VAMJEA	1D	7.5%	A	3.195	10.1002/zaac.201100148[28]
VUJRAW	0D	12.8%	A	3.416	CSD Communication (2020)
XODKEH	3D	0.2%	A	3.453	10.1021/ic701346x[29]

*HULTIT features a 1D polymer formed from the Copper sulfate dimer, and a separate 2D net formed from bridging sulfate groups. **Coordinates for HULTIT01 are found in refcode HULTIT.

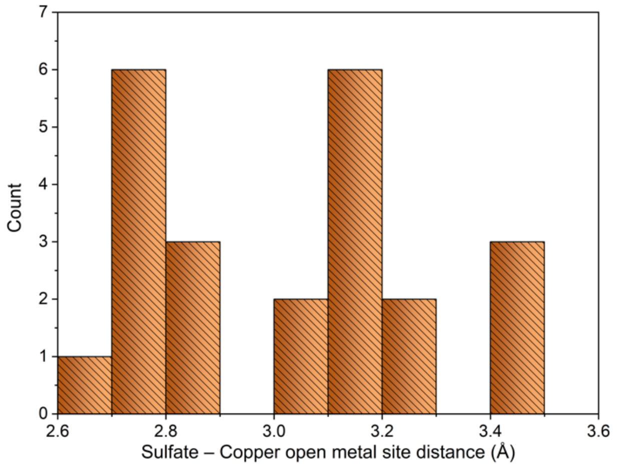

Figure S4: Histogram showing long sulfate-Cu bond length in $CuSO_4$ MBB dimer structures. A bimodal distribution is seen below 3.3 Å indicating the bimodal structural variation in MBB. Structures with long sulfate-Cu bond length above 3.3 Å are characterized by other functional groups blocking the copper binding site.

3 $CuSO_4(1,4\text{-bib})_{1.5}$, 1

3.1 Crystal structure of 1

Table S2: Structural features of 1 at 100 K, RT, 333 K, and 343 K.

	1op at 100 K	1op at RT	1op at 333 K	1cp at 343 K
**Copper sulfate MBB bond lengths**	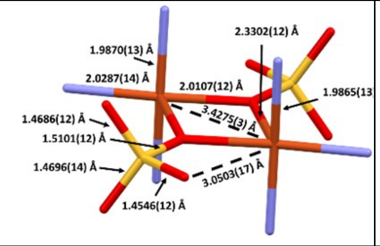	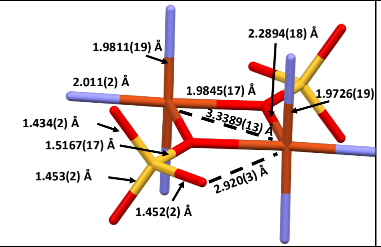	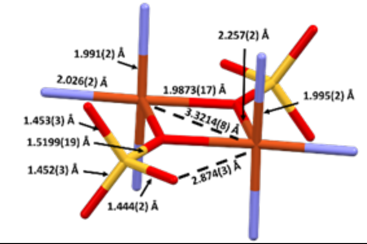	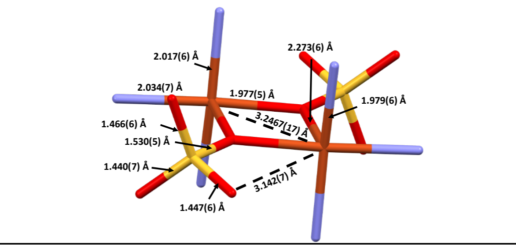
**Copper sulfate MBB bond angles**	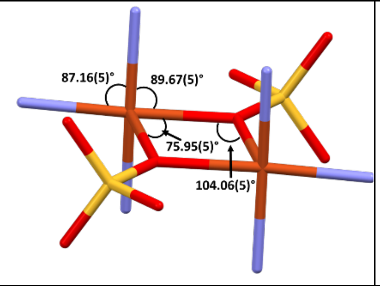	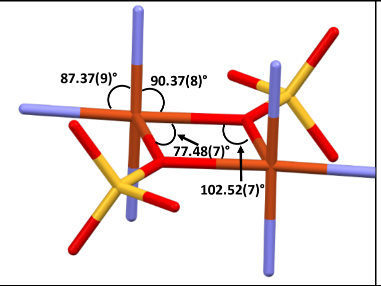	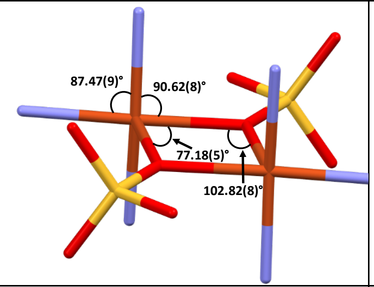	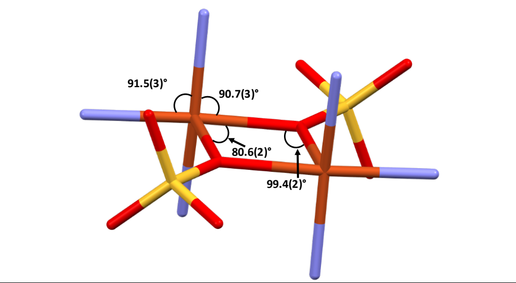
**Sql network dimensions**	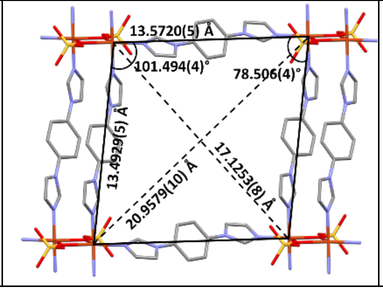	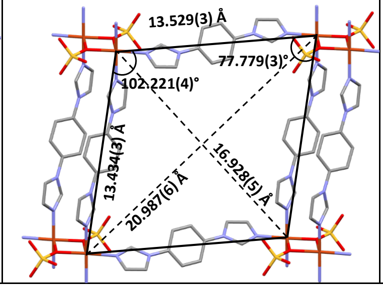	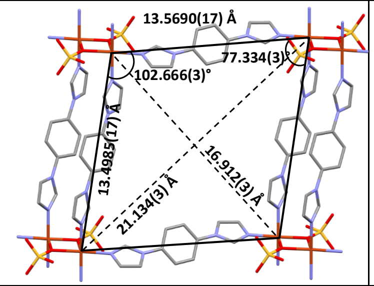	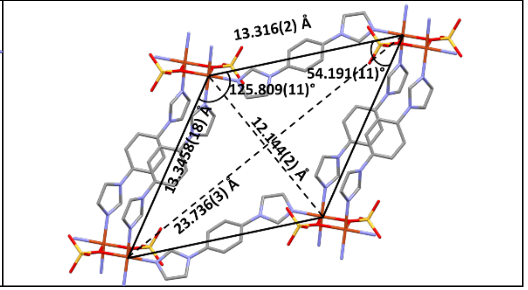

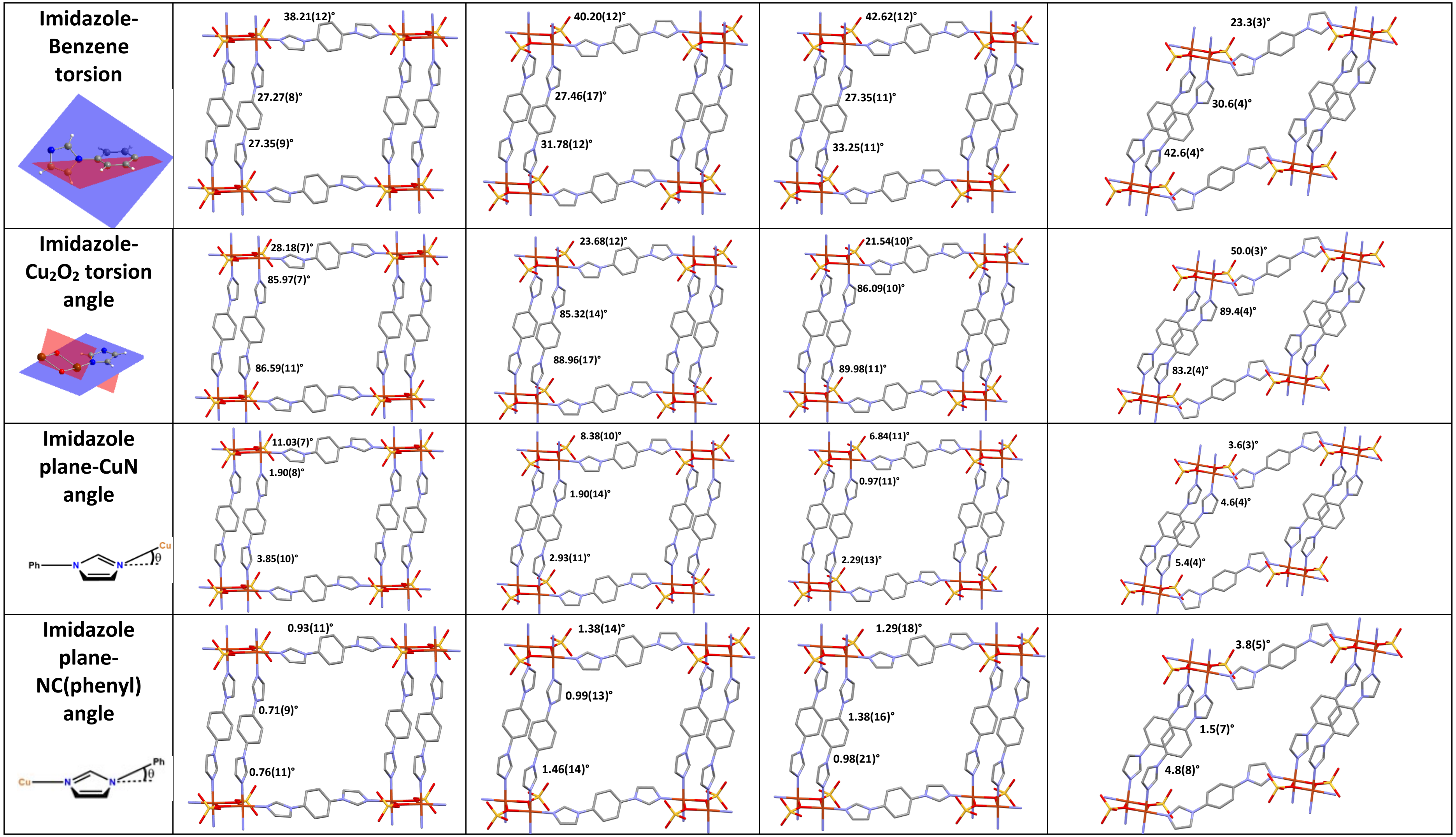
Imidazole-Benzene torsion
38.21(12)°
27.27(8)°
27.35(9)°
40.20(12)°
27.46(17)°
31.78(12)°
42.62(12)°
27.35(11)°
33.25(11)°
23.3(3)°
30.6(4)°
42.6(4)°
Imidazole-Cu2O2 torsion angle
28.18(7)°
85.97(7)°
86.59(11)°
23.68(12)°
85.32(14)°
88.96(17)°
21.54(10)°
86.09(10)°
89.98(11)°
50.0(3)°
89.4(4)°
83.2(4)°
Imidazole plane-CuN angle
Ph
N
N
Cu
θ
11.03(7)°
1.90(8)°
3.85(10)°
8.38(10)°
1.90(14)°
2.93(11)°
6.84(11)°
0.97(11)°
2.29(13)°
3.6(3)°
4.6(4)°
5.4(4)°
Imidazole plane-NC(phenyl) angle
Cu
N
N
Ph
θ
0.93(11)°
0.71(9)°
0.76(11)°
1.38(14)°
0.99(13)°
1.46(14)°
1.29(18)°
1.38(16)°
0.98(21)°
3.8(5)°
1.5(7)°
4.8(8)°

**Hydrogen bonding with O5**	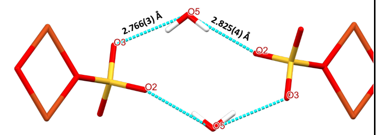	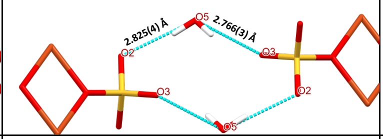	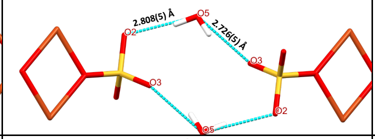	**–**
**Pore and void space**	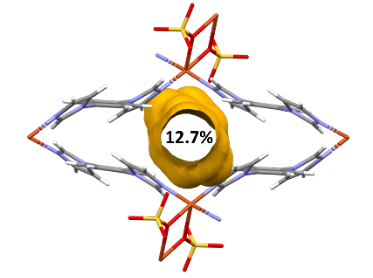	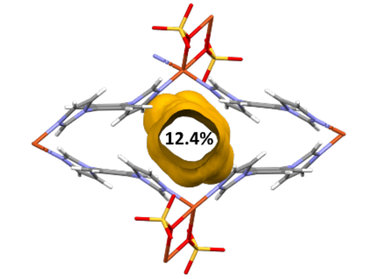	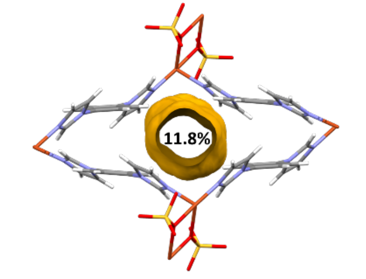	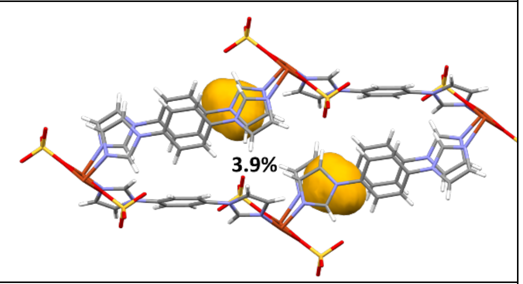
**Sulfate-Sulfate distance across pore**	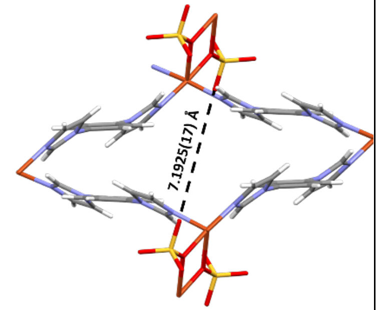	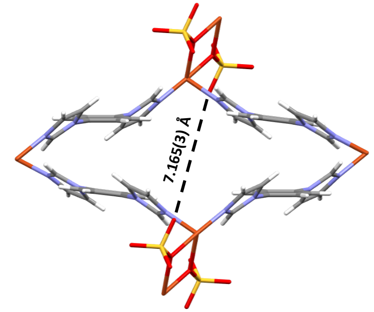	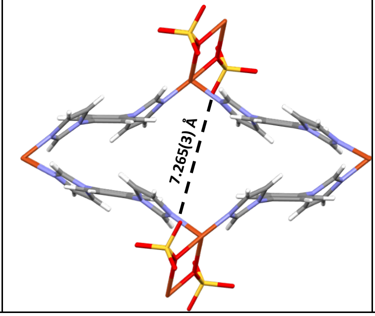	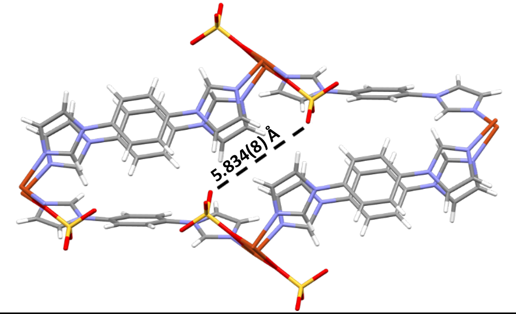

3.2 Thermogravimetric analysis (TGA) of 1op

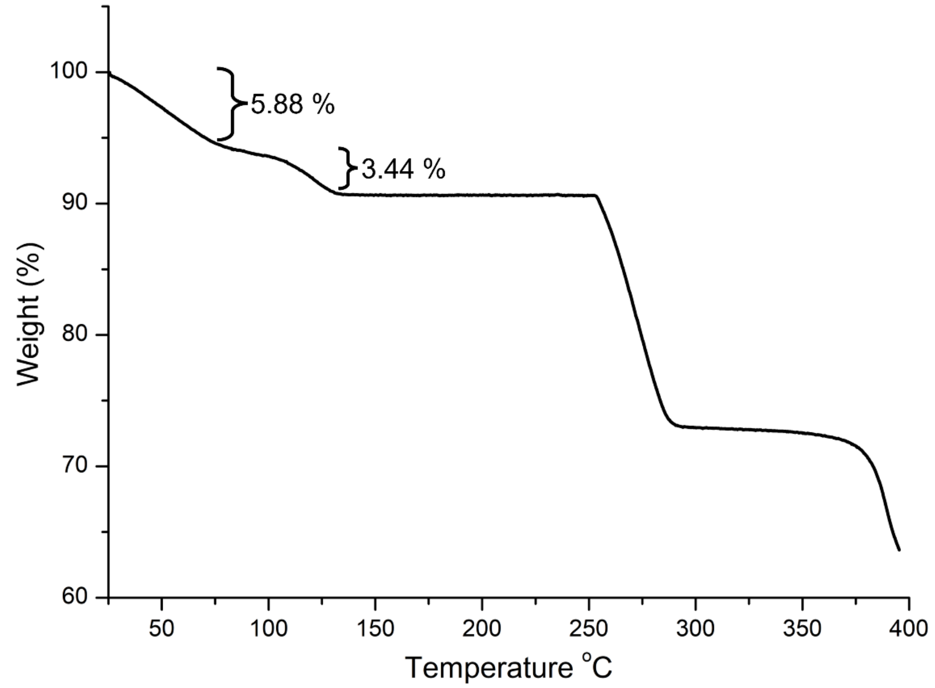

Figure S5: Thermogravimetric analysis of **1op**. Observed mass loss over two water loss events: 9.32%, expected mass loss: 9.21%. (based on $CuSO_4(1,4\text{-bib})_{1.5}\cdot 2.448(H_2O)$). Thermal degradation occurs in two steps beginning 250 °C and 370 °C.

3.3 Differential scanning calorimetry (DSC) for 1op

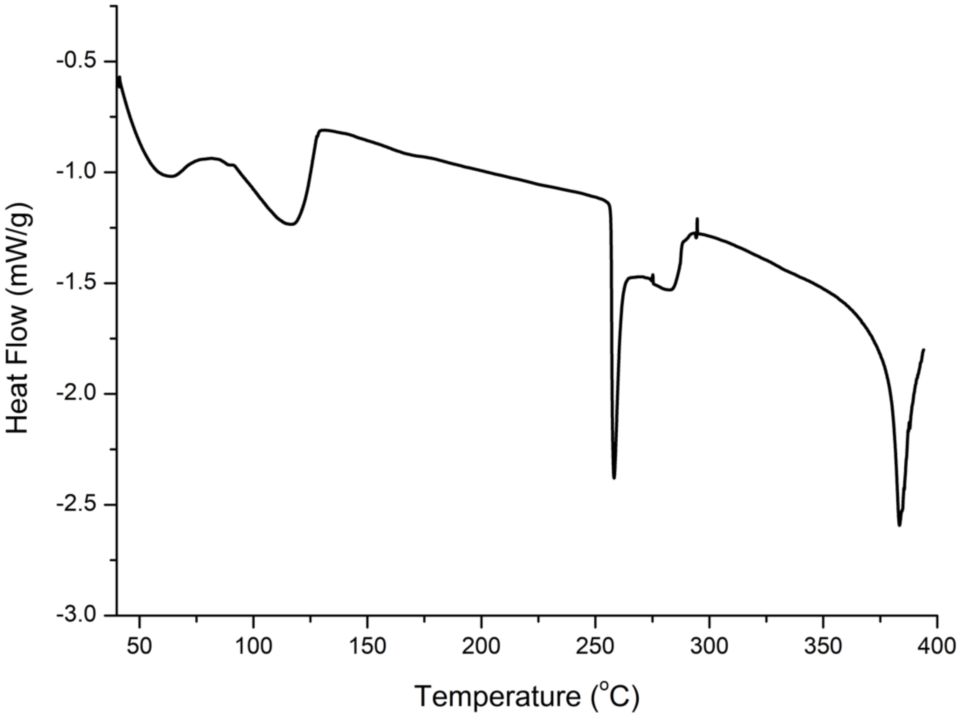

Figure S6: DSC trace of **1op**. Two peaks observed below 150 °C corresponding to water loss events, followed by thermal decomposition events beginning at 258 °C and 370 °C, matching mass loss observed in TGA.

3.4 Crystallographic information for 1

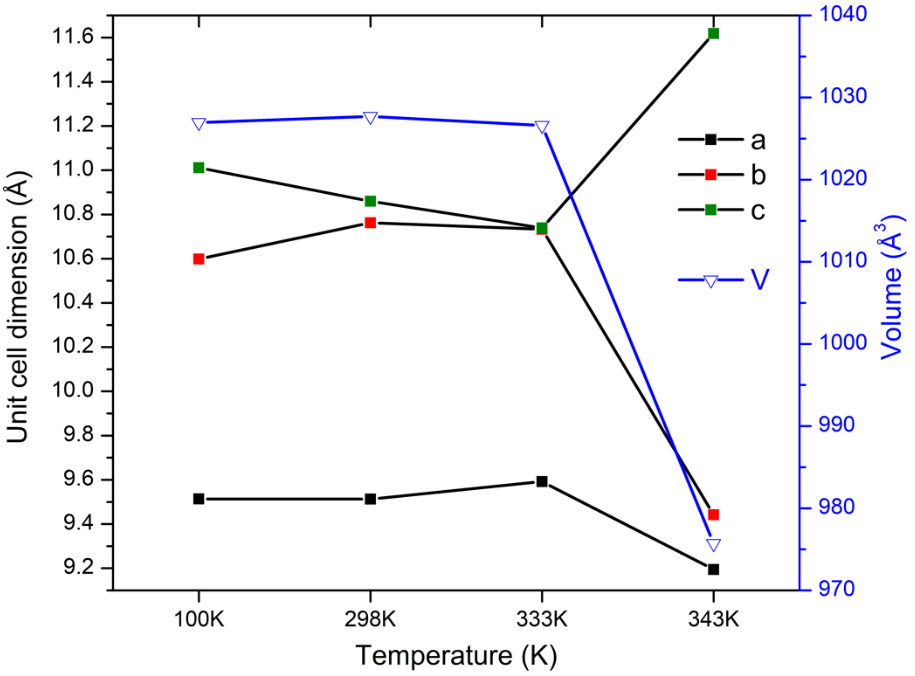

Figure S7: Unit cell dimensions (left) and volume (right) of **1** when collected at 100 K, 298 K, 333 K, and 343 K.

Table S3. Selected Crystallographic Data and Structure Refinement Parameters

**Compound**		**$CuSO_4(1,4\text{-bib})_{1.5}$, 1**		
Formula	$C_{18}H_{15}CuN_6O_4S$, 2.448(H_2O)	$C_{18}H_{15}CuN_6O_4S$, 1.263(H_2O)	$C_{18}H_{15}CuN_6O_4S$, 0.692(H_2O)	$C_{18}H_{15}CuN_6O_4S$
MW (g·mol^{-1})	522.69	497.66	487.40	474.96
T (K)	100(2)	299(2)	333(2)	343(2)
Crystal system	Triclinic	Triclinic	Triclinic	Triclinic
Space group	*P-1*	*P-1*	*P-1*	*P-1*
a (Å)	9.5137(4)	9.513(3)	9.5921(15)	9.1945(12)
b (Å)	10.5987(5)	10.762(4)	10.7331(17)	9.4416(13)
c (Å)	11.0115(5)	10.860(3)	10.7389(19)	11.6178(14)
α (°)	75.094(1)	76.558(10)	78.927(6)	89.907(5)
β (°)	81.849(1)	82.223(9)	71.470(5)	78.826(5)
γ (°)	73.705(2)	72.292(10)	83.027(5)	80.600(6)
V (Å^3)	1026.95(8)	1027.7(6)	1026.6(3)	975.7(2)
ρ_{calc} (g·cm^{-3})	1.690	1.608	1.577	1.617
Z, Z'	2, 1	2, 1	2, 1	2, 1
Observed reflections	5110	5191	3623	3426
R_1, wR_2 [I > 2σ(I)]	0.0285, 0.0688	0.0390, 0.1004	0.0365, 0.0932	0.0796, 0.1803
R_1, wR_2 (all data)	0.0335, 0.0712	0.0547, 0.1082	0.0471, 0.0988	0.1179, 0.2008
Goodness-of-fit on F^2	1.030	1.078	1.046	1.095
R_{int} value (%)	2.91	4.25	9.72	9.66
CCDC number	2288637	2288638	2288639	2288640

3.5 Overlay of 1op at 100 K and 1cp at 343 K

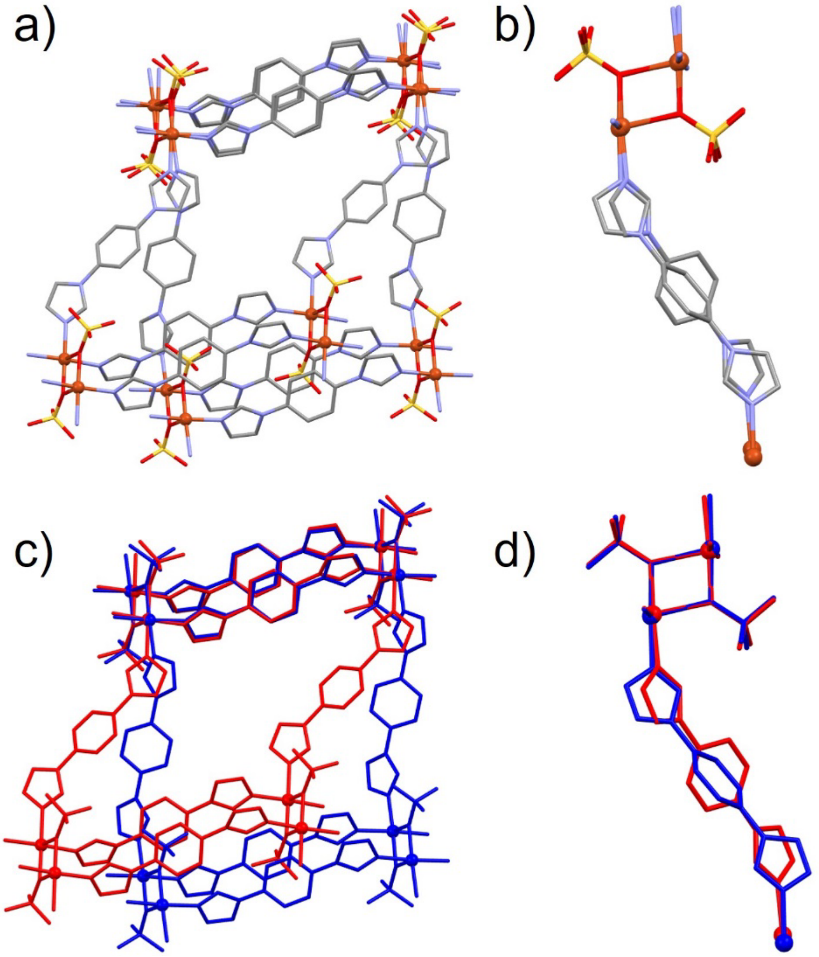

Figure S8: Structural overlay of **1op** at 100 K and **1cp** at 343 K formed by overlaying the Cu_2O_2 of each structure: a) **sql** net, b) $CuSO_4$ MBB dimer and the single-wall 1,4-bib ligand, c) **sql** net with **1op** at 100 K shown in blue and **1cp** at 343 K in red, d) $CuSO_4$ MBB dimer and the single-wall 1,4-bib ligand with **1op** at 100 K shown in blue and **1cp** at 343 K in red. Hydrogen atoms and solvated water molecules have been omitted for clarity.

3.6 Variable Temperature Powder X-ray diffraction (VTPXRD) of 1

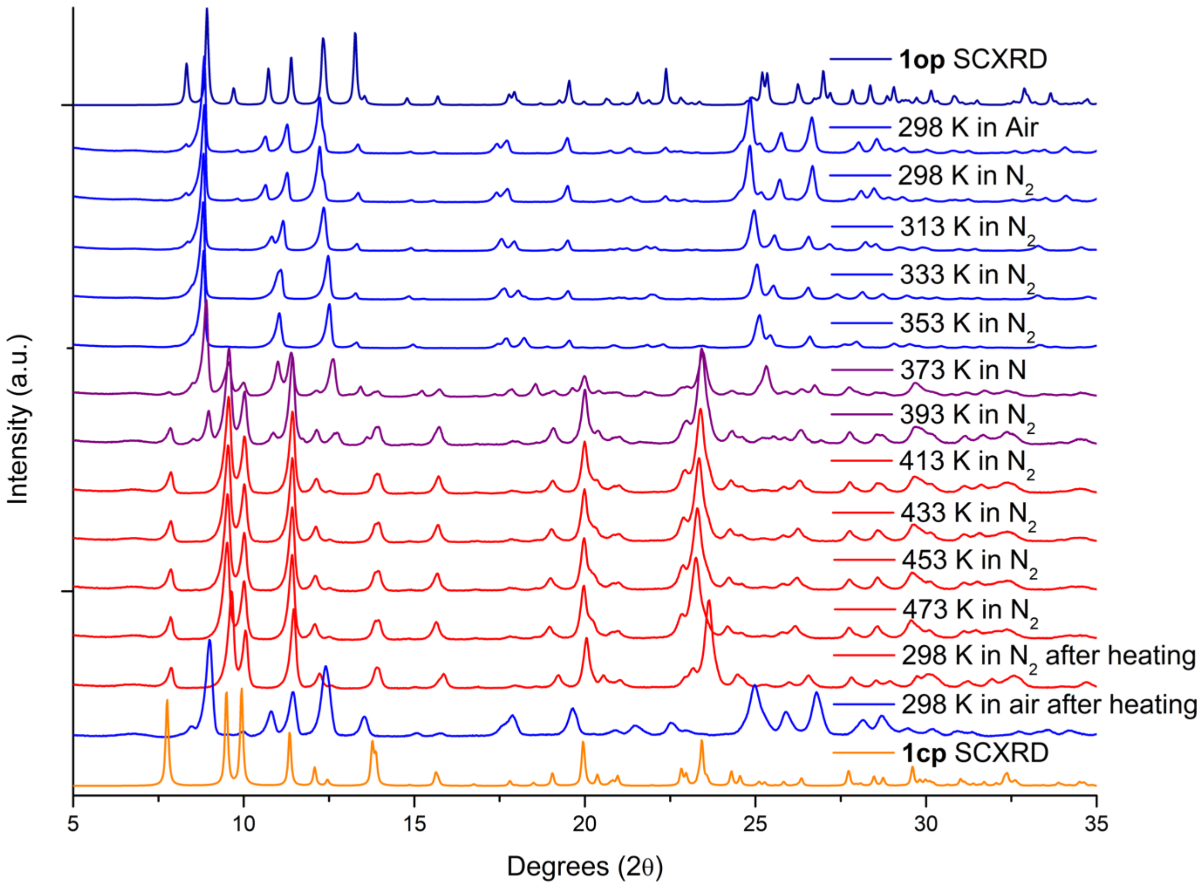

Figure S9: An overlay of the calculated PXRD of **1** collected at 100 K with the experimental PXRD of the as synthesized **1op** material at 298 K, followed by the material under dry N_2 at 298 K, 313 K, 333 K, 353 K, 373 K, 393 K, 413 K, 433 K, 453 K, 473 K. The sample was then allowed to cool to 298 K while remaining under dry N_2 then exposed to air for 5 min. From this it can be seen that the open phase slowly changes as the temperature rises, then at 373 K the **1cp** begins to appear and is the only phase present at 413 K up to 473 K. When the sample is cooled to 298 K the closed phase persists, and when exposed to air the sample reverts to **1op**, as would be indicated by the water sorption of the sample. Each PXRD is colored to match the phase with the **1op** calculated PXRD in Navy blue, and experimental PXRD patterns in blue, **1cp** calculated PXRD in orange, and experimental PXRD patterns in red. PXRD patterns where a mixture of **1op** and **1cp** is present are shown in purple.

3.7 Dynamic vapor sorption (DVS) of 1

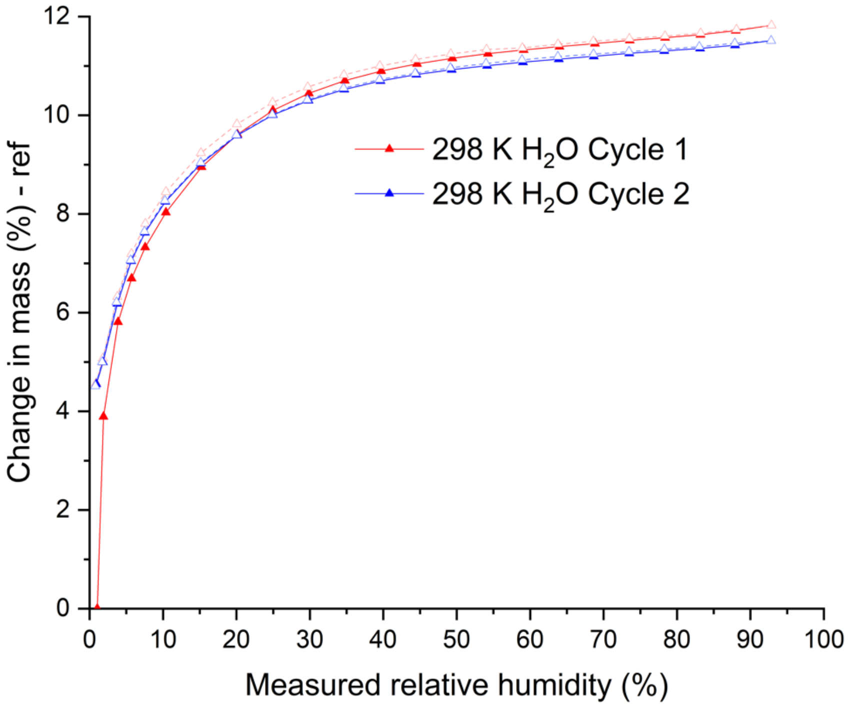

Figure S10: Dynamic vapor sorption experiment showing water sorption and desorption of a 100 °C activated sample of **1** at 298 K (red). A second cycle (blue) was then performed without reactivation where the change in mass was normalized using the mass at the beginning of cycle 1. From this it can be seen that the strongly bound water molecule does not fully desorb in the first cycle at RT, and so cycle 2 begins with strongly bound water already present in the material.

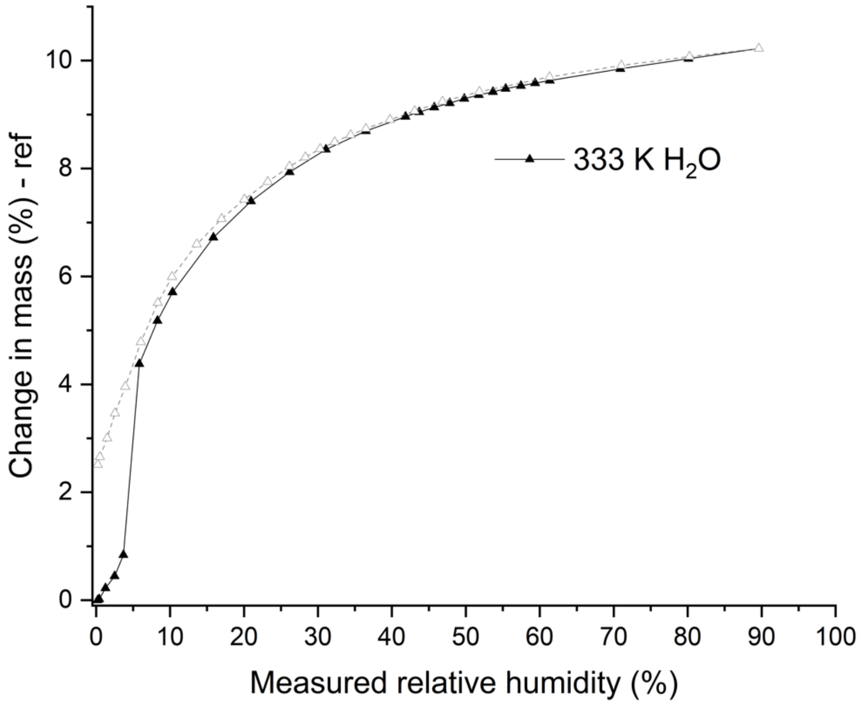

Figure S11: H_2O dynamic vapor sorption of **1** at 333 K of a 100 °C activated sample. At *ca.* 5% RH a sharp rise in uptake is observed, indicating a switch from **1cp** to **1op** structures.

3.8 High Pressure CO_2 sorption of 1

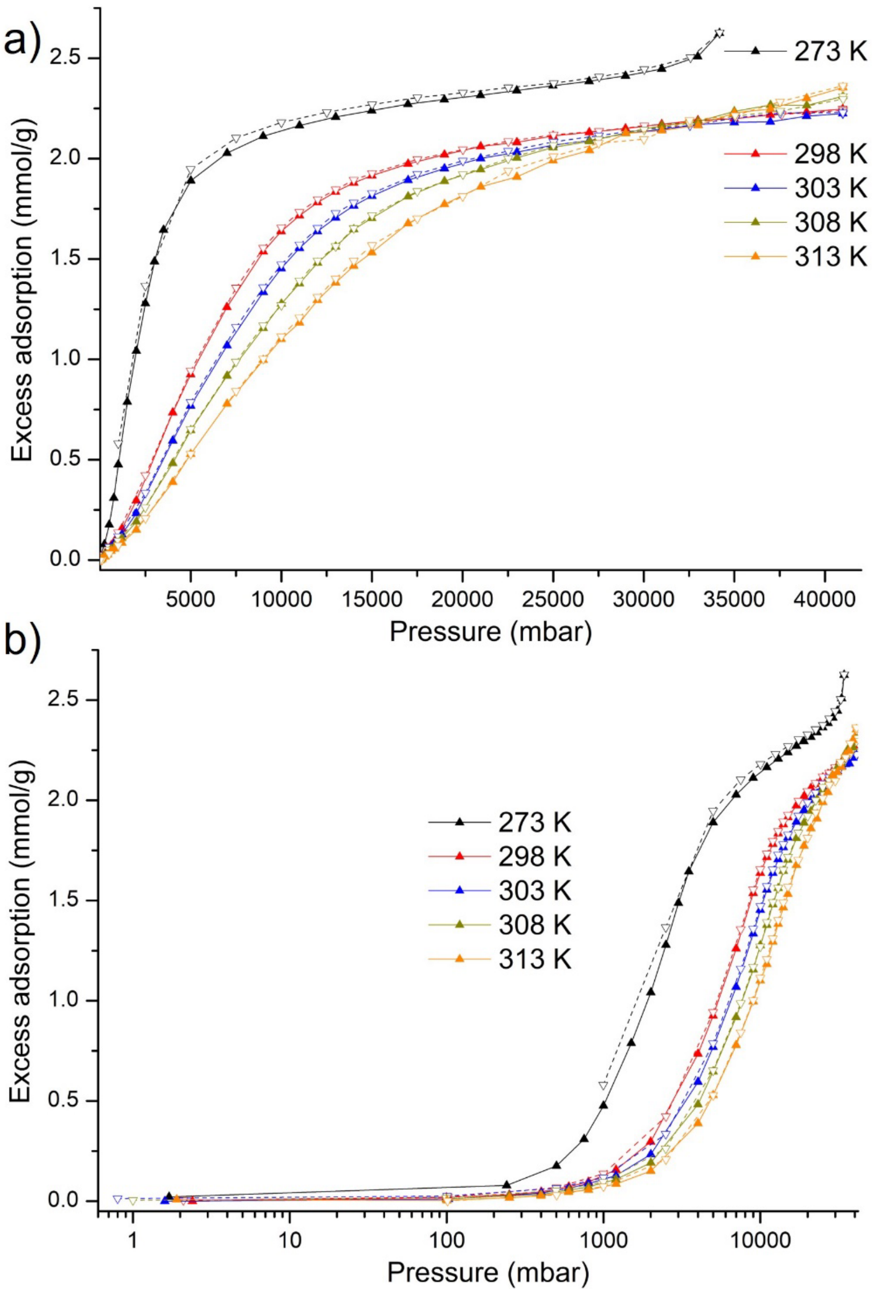

Figure S12: High pressure CO_2 sorption of **1** showing a type F-III isotherm at 273 K, 298 K, 303 K, 308 K, and 313 K on a) linear scale and b) logarithmic scale.

3.9 Low pressure CO_2, C_2H_2, C_2H_4, C_2H_6 sorption at 298 K of 1

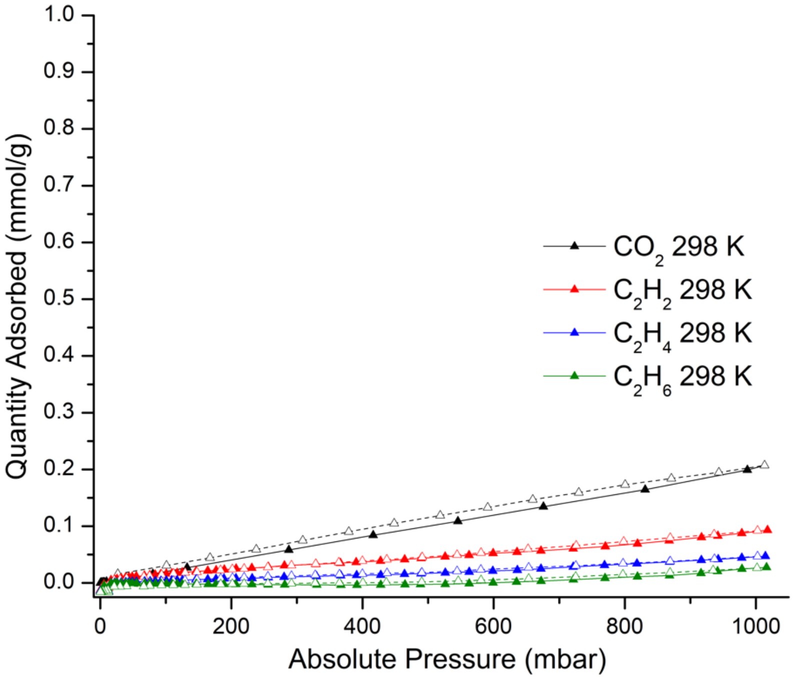

Figure S13: 298 K sorption of **1** for CO_2 and C2 hydrocarbon gases showing no appreciable uptake.

3.10 RT-activated 298 K CO_2 sorption of 1op

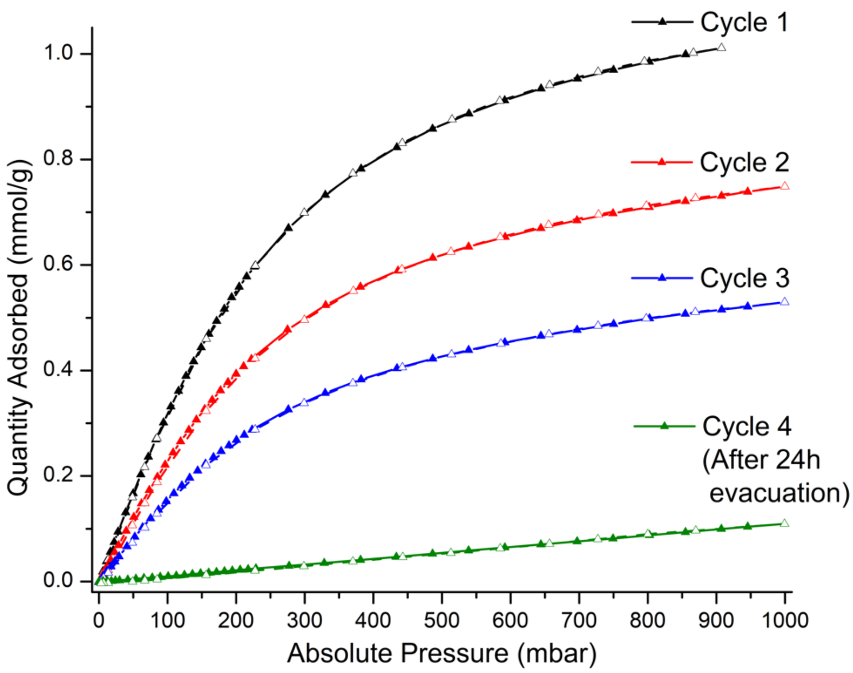

Figure S14: 298 K CO_2 sorption of **1** activated at RT directly within the gas sorption instrument. Successive cycles show a decline in uptake as a result of slow conversion of **1op** to **1cp**.

3.11 Slurry-based synthesis of 1

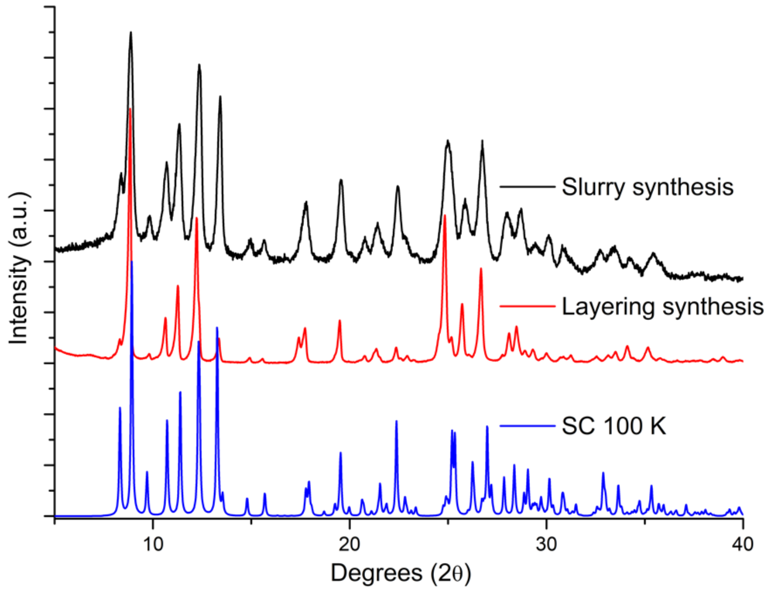

Figure S15: An overlay of the experimental PXRD of **1op** synthesized from a H_2O:MeOH slurry of 1,4-bib and $CuSO_4{\cdot}5H_2O$, with the experimental PXRD of **1op** formed from layering and the calculated PXRD of a single crystal of **1op** collected at 100 K.

3.12 PXRD after thermal decomposition of 1

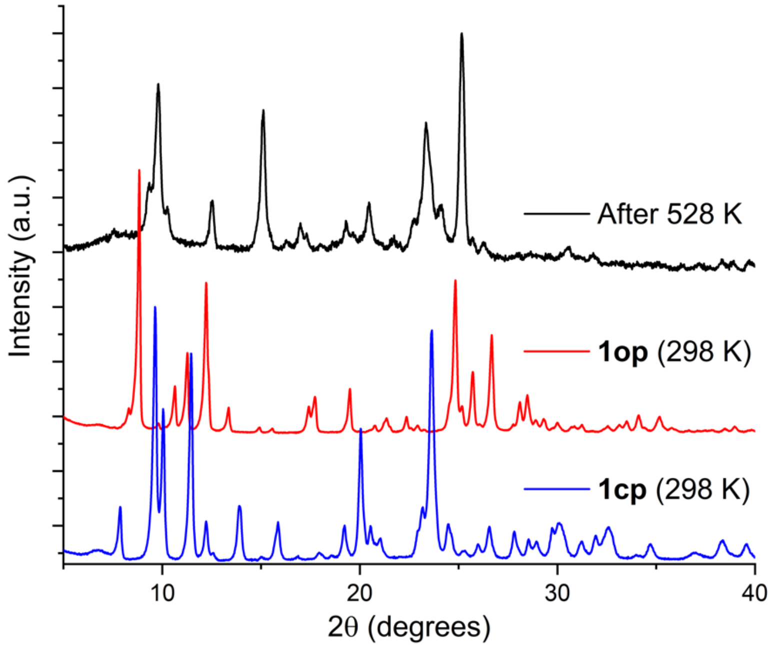

Figure S16: An overlay of the experimental PXRD of **1** collected after heating a sample to 528 K. As seen in the TGA and DSC plots, the material thermally decomposes at this temperature and so the PXRD of the material changes. Experimental PXRD patterns of **1op** and **1cp** collected at 298 K are shown for comparison.

4 $CuSO_4(1,4\text{-bin})_{1.5}$, 2

4.1 Crystal structure of 2

Table S4: Structural features of 2op and 2np at 100 K.

Note: Occupancy for the minor component of 2op – 100 K is low (14.5%), and 2np – 100 K (major and minor components) have poor data quality and so are heavily restrained. Therefore, measurements taken from these phases will be less accurate than indicated by estimated standard deviation and should be treated with extreme caution. Disorder of the single-ligand wall naphthalene rings are omitted for clarity.

	2op - Major component (85.5%)	2op - Minor component (14.5%)	2np - Major component (75%)	2np - Minor component (25%)
Copper sulfate MBB bond lengths	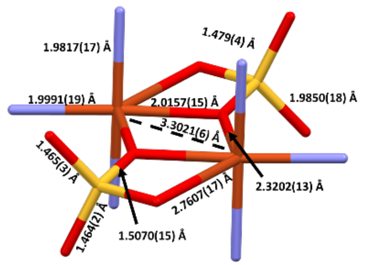	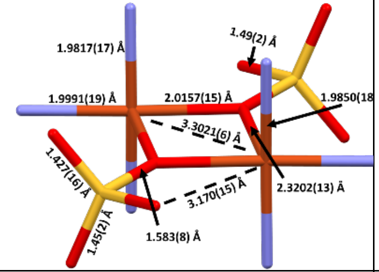	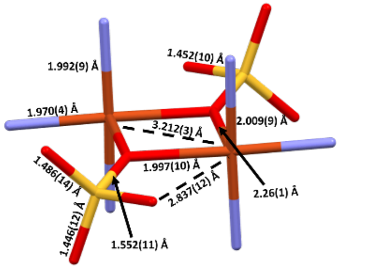	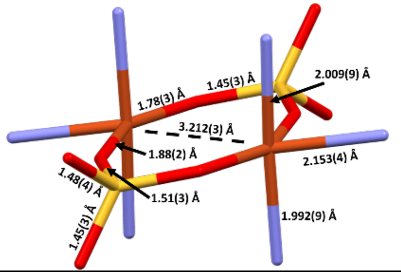
Copper sulfate MBB bond angles	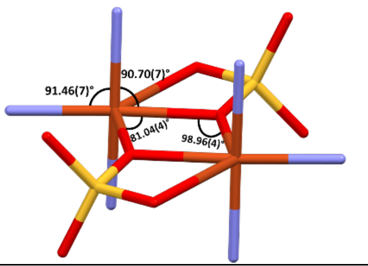	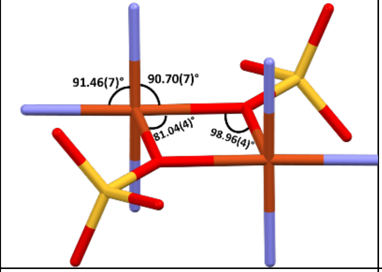	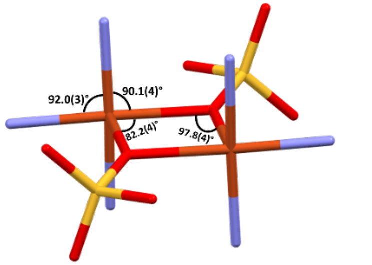	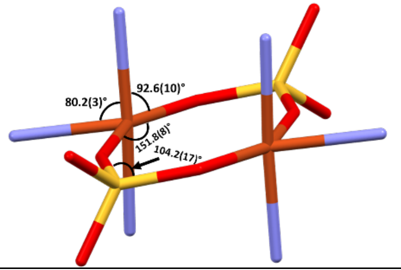
Sql network dimensions	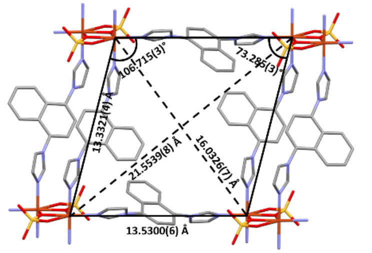	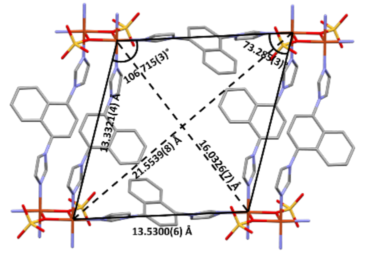	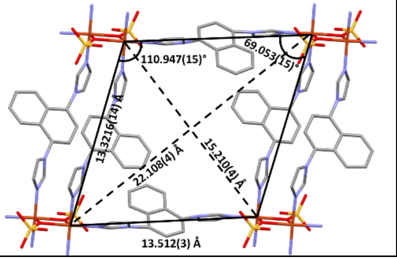	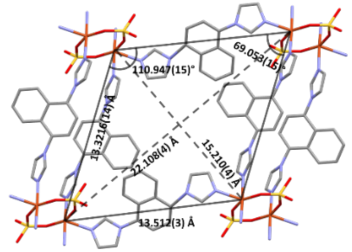

<table>
<tr><td>Imidazole-Benzene torsion
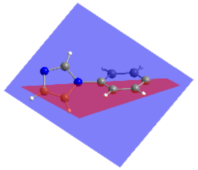</td>
<td>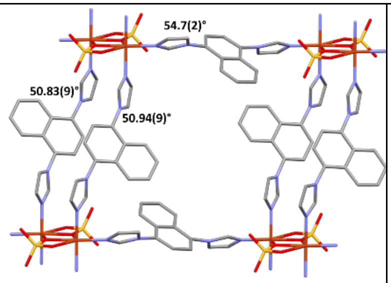
</td>
<td>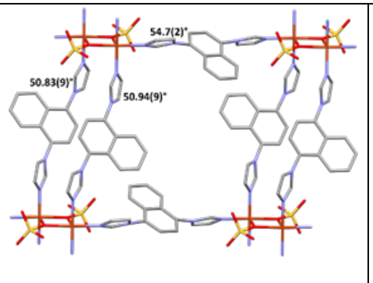
</td>
<td>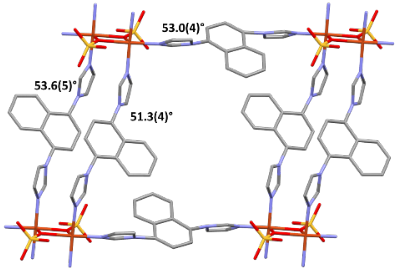
</td>
<td>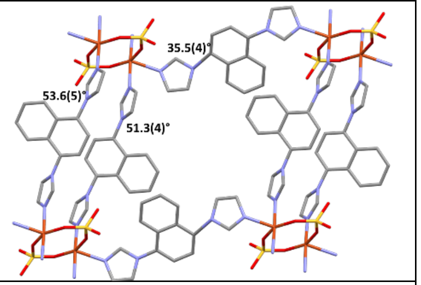
</td></tr>
<tr><td>Imidazole-Cu_2O_2 (Or $Cu_2(SO_2)_2$ for activated phase minor component) torsion angle
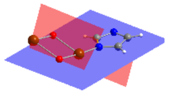</td>
<td>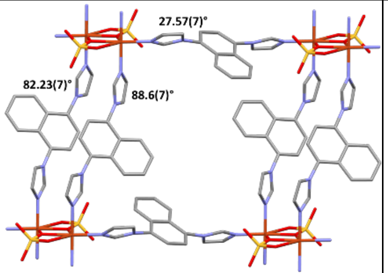
</td>
<td>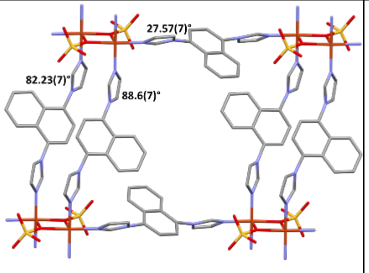
</td>
<td>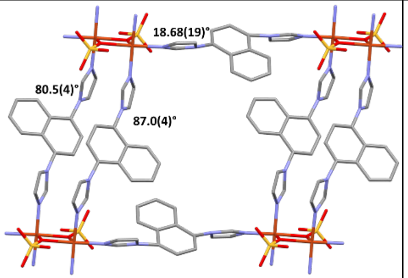
</td>
<td>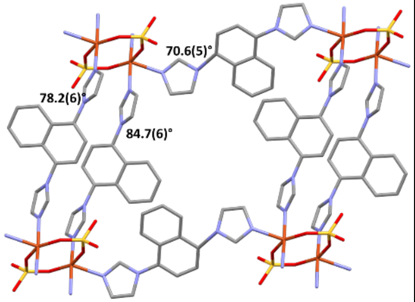
</td></tr>
<tr><td>Imidazole plane-CuN angle
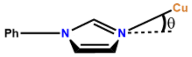
</td>
<td>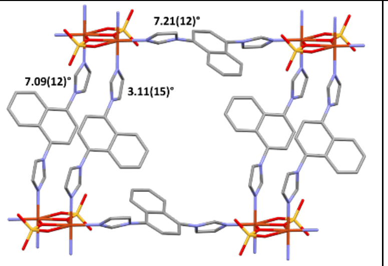
</td>
<td>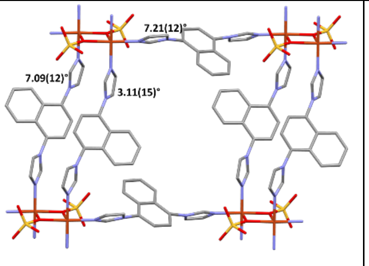
</td>
<td>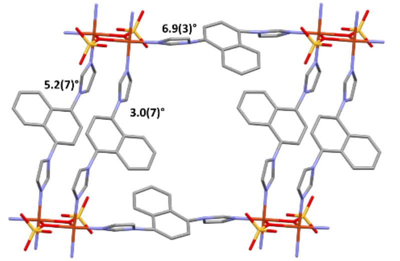
</td>
<td>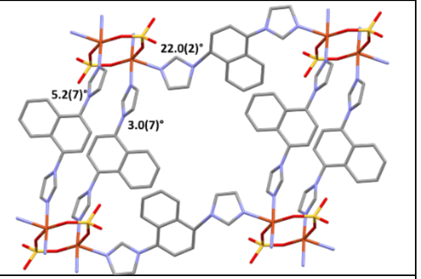
</td></tr>
<tr><td>Imidazole plane-NC(phenyl) angle
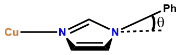
</td>
<td>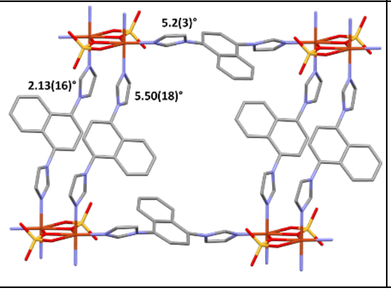
</td>
<td>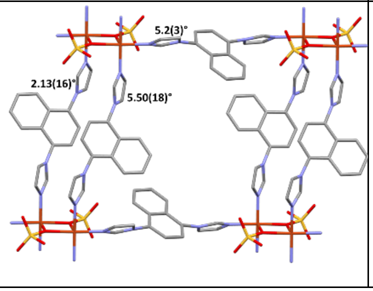
</td>
<td>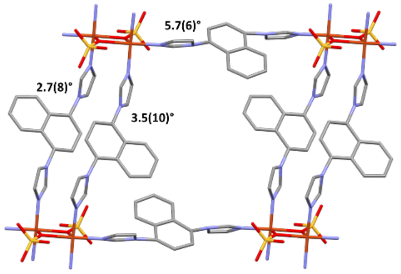
</td>
<td>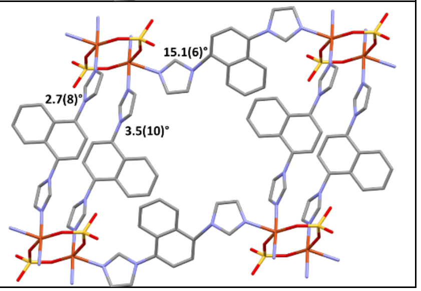
</td></tr>
</table>

Table S5: Structural features of 2op and 2np at RT.

Note: Occupancy for the minor component of 2op-RT is low (14.4%), and 2np-RT (major and minor components) have poor data quality and so are heavily restrained. Therefore, measurements taken from these phases will be less accurate than indicated by estimated standard deviation and should be treated with extreme caution. Disorder of the single-ligand wall naphthalene rings are omitted for clarity.

	2op - Major component (85.6%)	2op - Minor component (14.4%)	2np - Major component (75%)	2np - Minor component (25%)
Copper sulfate MBB bond lengths	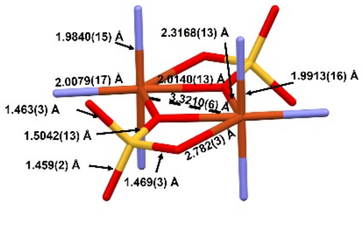	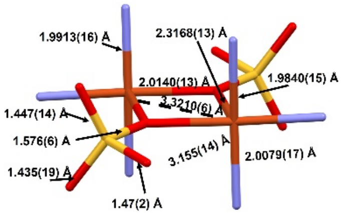	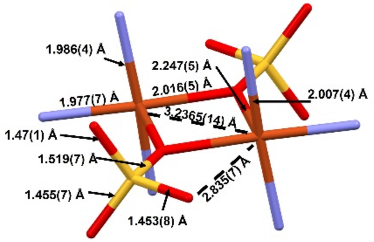	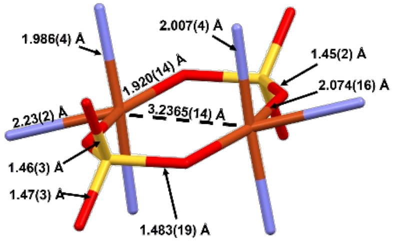
Copper sulfate MBB bond angles	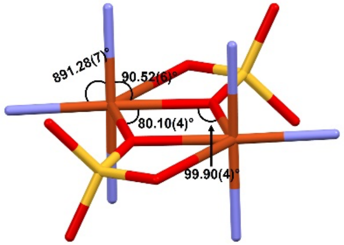	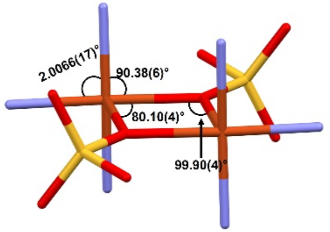	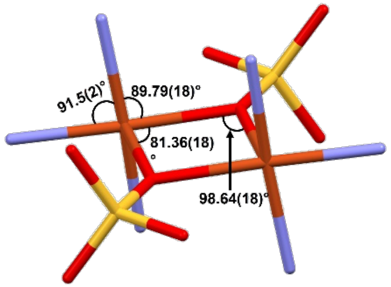	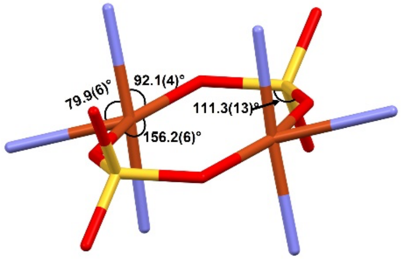
Sql network dimensions	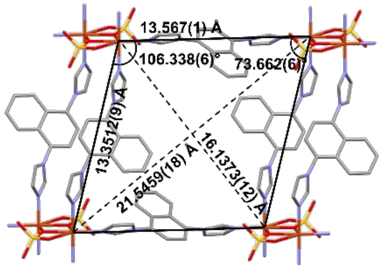	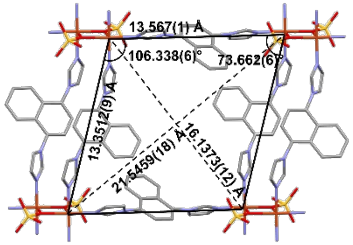	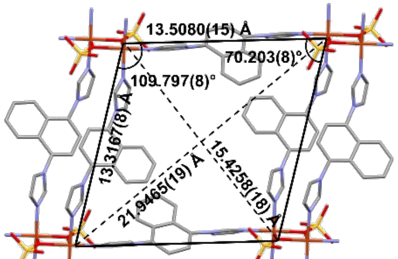	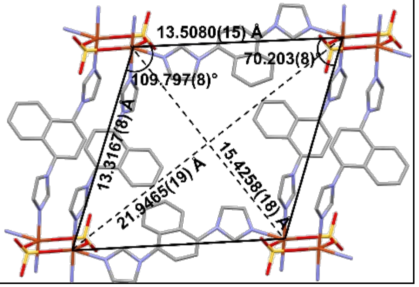

**Imidazole-Benzene torsion**<br>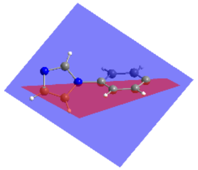	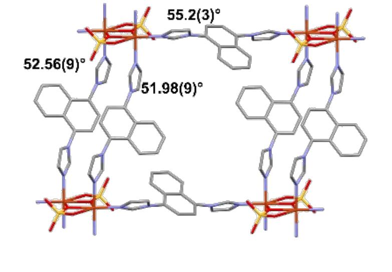	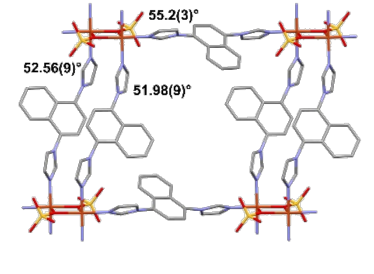	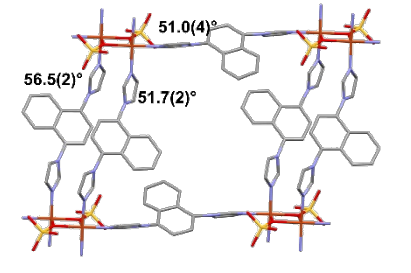	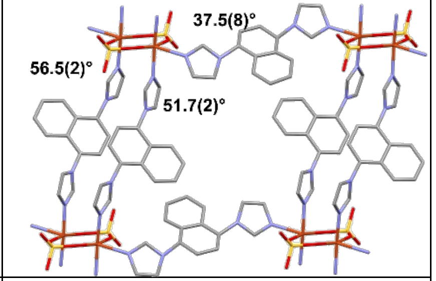
**Imidazole-Cu_2O_2 (Or $Cu_2(SO_2)_2$ for activated phase minor component) torsion angle**<br>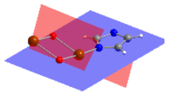	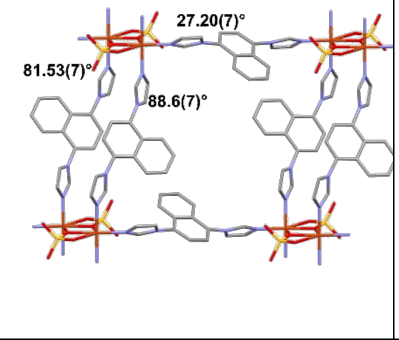	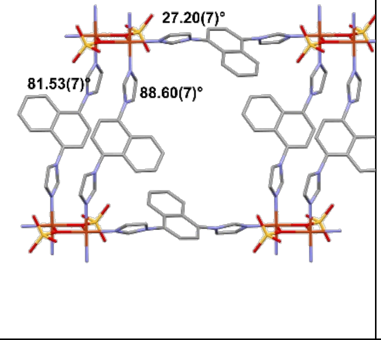	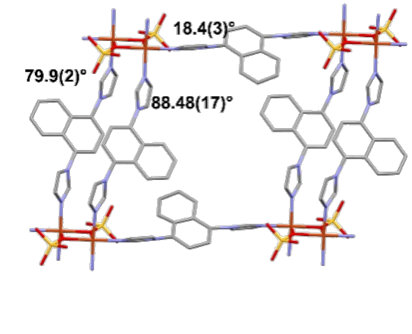	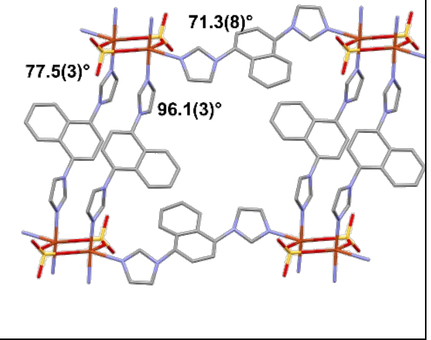
**Imidazole plane-CuN angle**<br>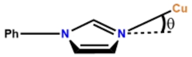	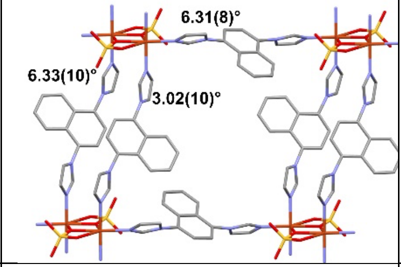	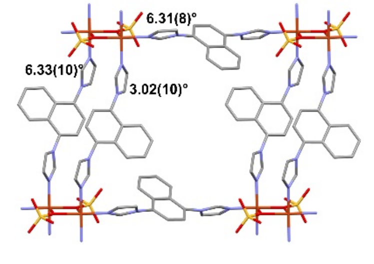	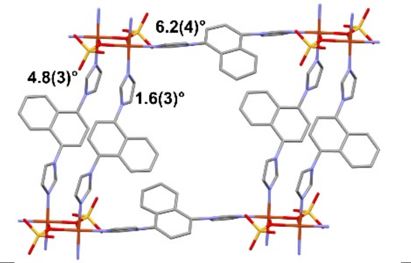	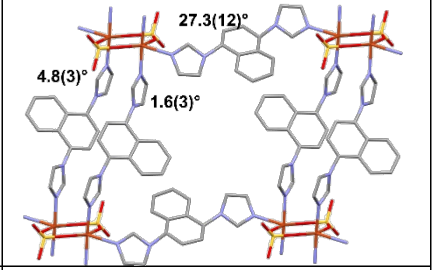
**Imidazole plane-NC(phenyl) angle**<br>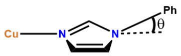	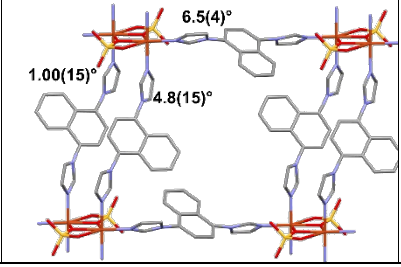	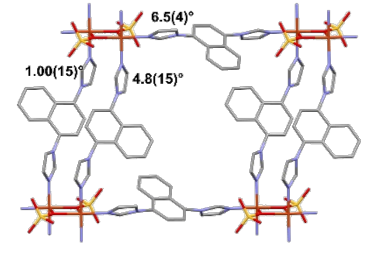	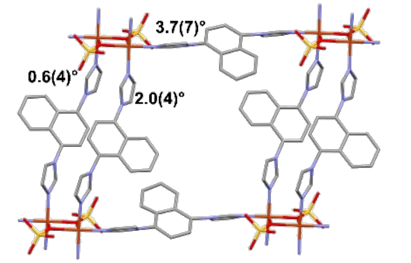	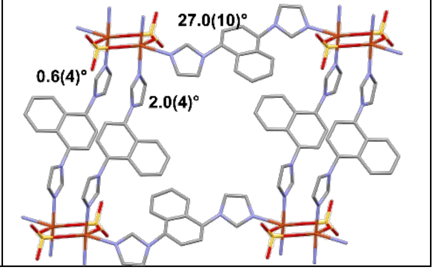

4.1 Overlay of 2op and 2np at 100 K

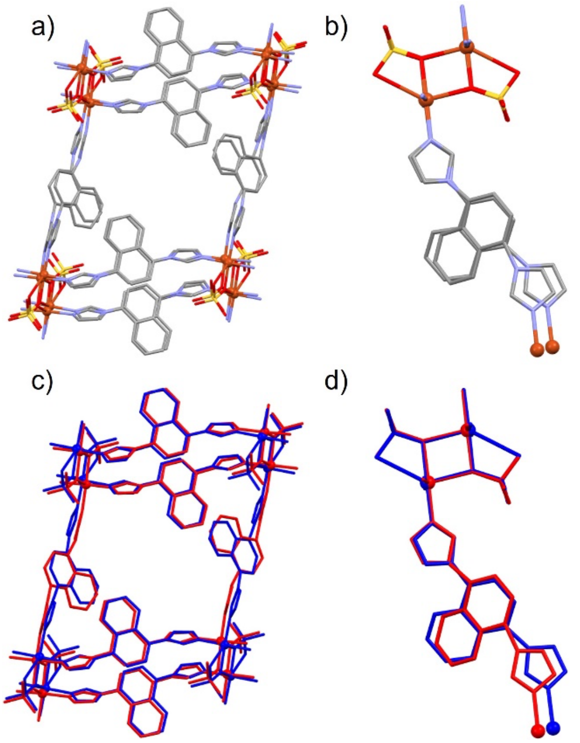

Figure S17: Structural overlay of the major components of **2op** and **2np** at 100 K formed by overlaying the Cu_2O_2 rings of each structure: a) **sql** net, b) $CuSO_4$ MBB dimer and the single-wall 1,4-bin ligand, c) **sql** net with **2op** shown in blue and **2np** in red, d) $CuSO_4$ MBB dimer and the single-wall 1,4-bin ligand with the **2op** shown in blue and **2np** in red. Hydrogen atoms, solvated water molecules, and disordered groups have been omitted for clarity.

Table S6. Selected Crystallographic Data and Structure Refinement Parameters

**Compound**	**$CuSO_4(1,4\text{-bin})_{1.5}$, 2**			
Formula	$C_{24}H_{28}CuN_6O_9S$	$C_{24}H_{28}CuN_6O_9S$	$C_{24}H_{18}CuN_6O_4S$	$C_{24}H_{18}CuN_6O_4S$
MW (g·mol^{-1})	640.12	640.12	550.04	550.04
T (K)	100.00	RT	100.00	298.00
Crystal system	triclinic	triclinic	triclinic	triclinic
Space group	P-1	P-1	P-1	P-1
a (Å)	11.3146(4)	11.4836(10)	9.2769(12)	9.2929(6)
b (Å)	11.6294(4)	11.6895(10)	11.9054(16)	12.0305(8)
c (Å)	13.1637(4)	13.2452(11)	12.0895(16)	12.1924(9)
α (°)	104.5870(10)	104.333(2)	100.430(5)	100.900(3)
β (°)	114.5060(10)	115.102(2)	104.128(4)	106.348(3)
γ (°)	100.6310(10)	100.508(3)	106.101(5)	103.911(3)
V (Å^3)	1440.82(8)	1473.9(2)	1198.9(3)	1220.42(15)
ρ_{calc} (g·cm^{-3})	1.475	1.442	1.524	1.497
Z, Z'	2, 1	2, 1	2, 1	2, 1
Observed reflections	7183	7308	4202	4331
R_1, wR_2 [$I > 2\sigma(I)$]	0.0340, 0.0946	0.0310, 0.0948	0.1175, 0.2966	0.0772, 0.2310
R_1, wR_2 (all data)	0.0375, 0.0968	0.0329, 0.0962	0.1549, 0.3347	0.0837, 0.2418
Goodness-of-fit on F^2	1.055	1.108	1.046	1.102
R_{int} value (%)	2.70	3.98	5.22	5.89
CCDC number	2288641	2331110	2288642	2331109

4.2 Thermogravimetric analysis (TGA) of 2op

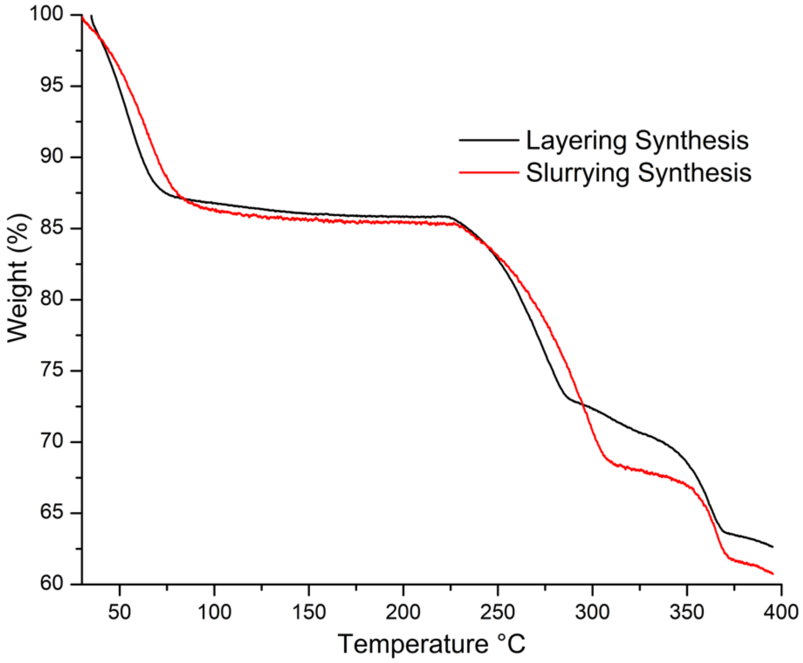

Figure S18: Thermogravimetric analysis of **2op** from layering and slurrying synthesis. Water loss occurs < 100 °C, followed by thermal degradation events beginning at 227 °C and > 340 °C.

4.3 Differential scanning calorimetry (DSC) for 2op

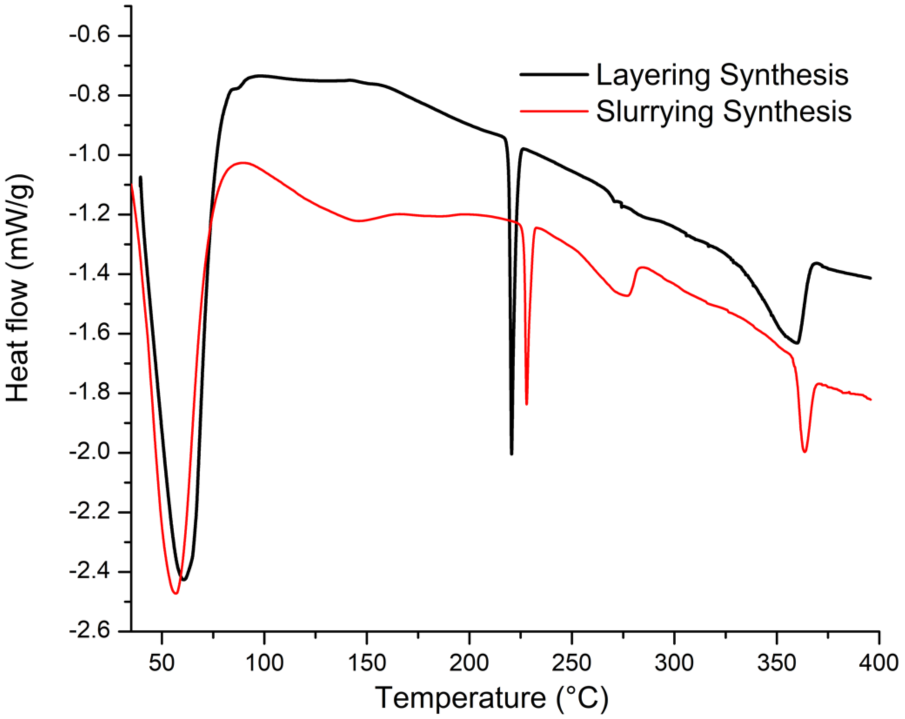

Figure S19: DSC trace of **2op** from layering and slurrying synthesis. Thermal event corresponding to water loss occurs < 100 °C, followed by thermal degradation events observed at 220 °C for layering and 227 °C for slurrying, then further degradation at 334 °C for layering and 358 °C for slurrying.

4.4 Variable Temperature Powder X-ray diffraction (VTPXRD) of 2

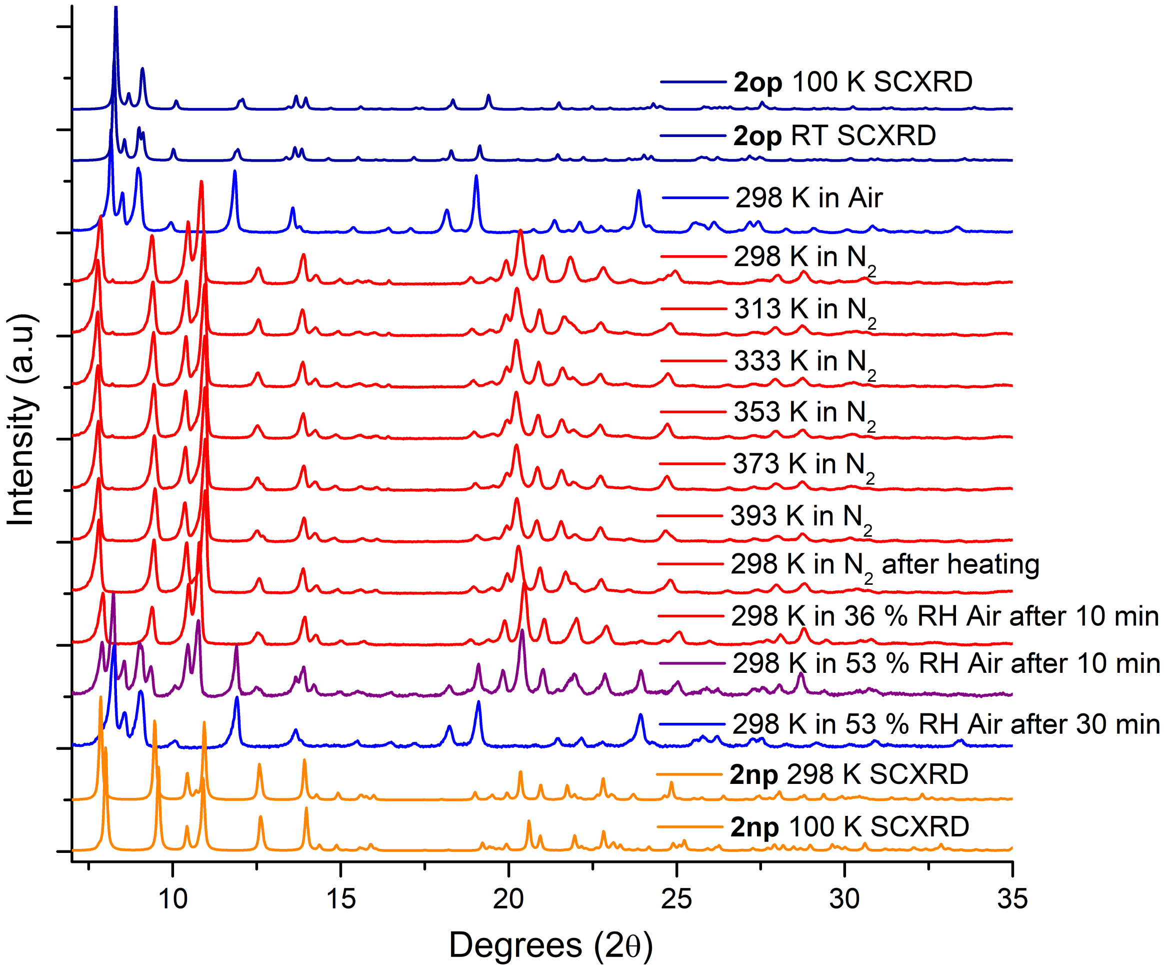

Figure S20: An overlay of the calculated PXRD of **2op** collected at 100 K and RT with the experimental PXRD of **2op** in air at 298 K, followed by the material under dry N_2 at 298 K, 313 K, 333 K, 353 K, 373 K, 393 K. The sample was then allowed to cool to 298 K while remaining under dry N_2 then exposed to 3% RH air for 10 min, then to 53% RH air for 10 min then to 53% RH air after a total time of 30 min. From this it can be seen that the **2op** phase immediately changes to the **2np** phase upon exposure to dry N_2 at 298 K and this phase is maintained as the temperature rises to 393 K, and as the sample is cooled to 298 K and exposed to 36% RH air for 10 min. When the sample is exposed to 53% RH air for 10 min, a mixture of **2op** and **2np** are observed, and after 30 min only **2op** is present. Each PXRD is colored to match the phase with **2op** (100 K and RT) calculated PXRD in Navy blue, and experimental PXRD patterns in blue, **2np** calculated PXRD in orange (100 K and 298 K), and experimental PXRD patterns in red. The PXRD pattern where a mixture of **2op** and **2np** is present is shown in purple.

4.5 PXRD, sorption and IAST selectivity for 2-Slurrying

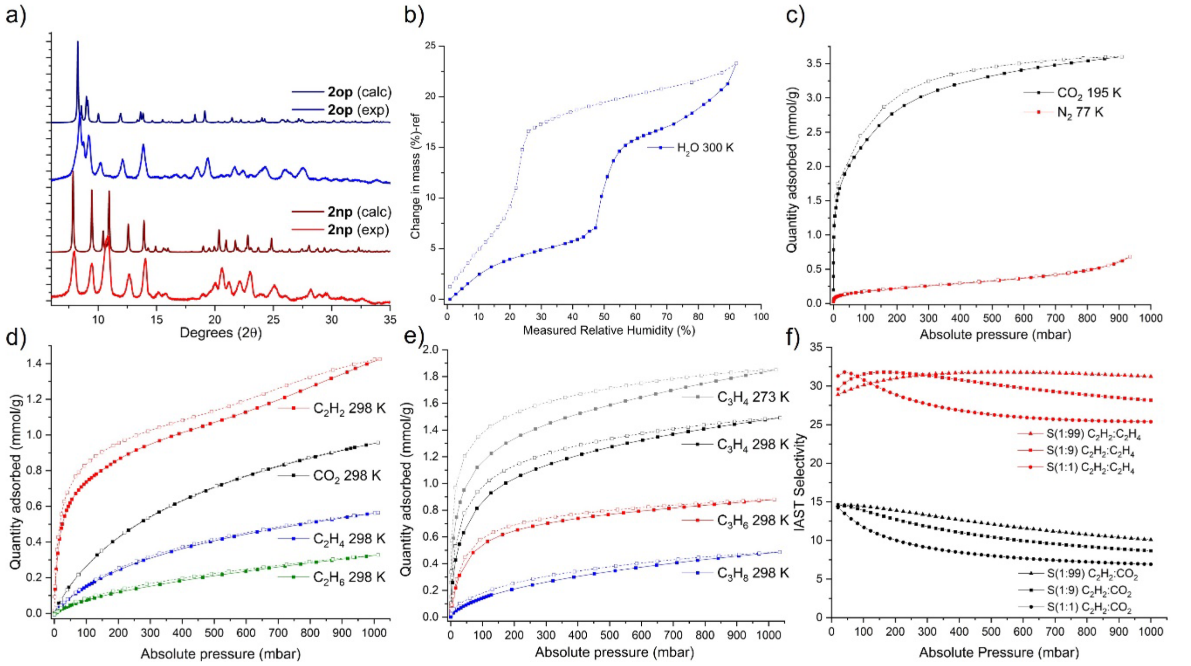

Figure S21: a) Overlay of calculated (at RT) and experimental PXRD patterns of **2** in the **2op** and **2np** phases when synthesized by slurrying; b) water vapor sorption at 300 K; c) CO_2 adsorption isotherm at 195 K; d) CO_2, C_2H_2, C_2H_4, and C_2H_6 adsorption isotherms at 298 K; e) adsorption isotherms for C_3H_4 at 273 K and 298 K, C_3H_6 and C_3H_8 at 298 K; f) C_2H_2/CO_2 and C_2H_2/C_2H_4 IAST selectivity at 298 K for various compositions vs. pressure.

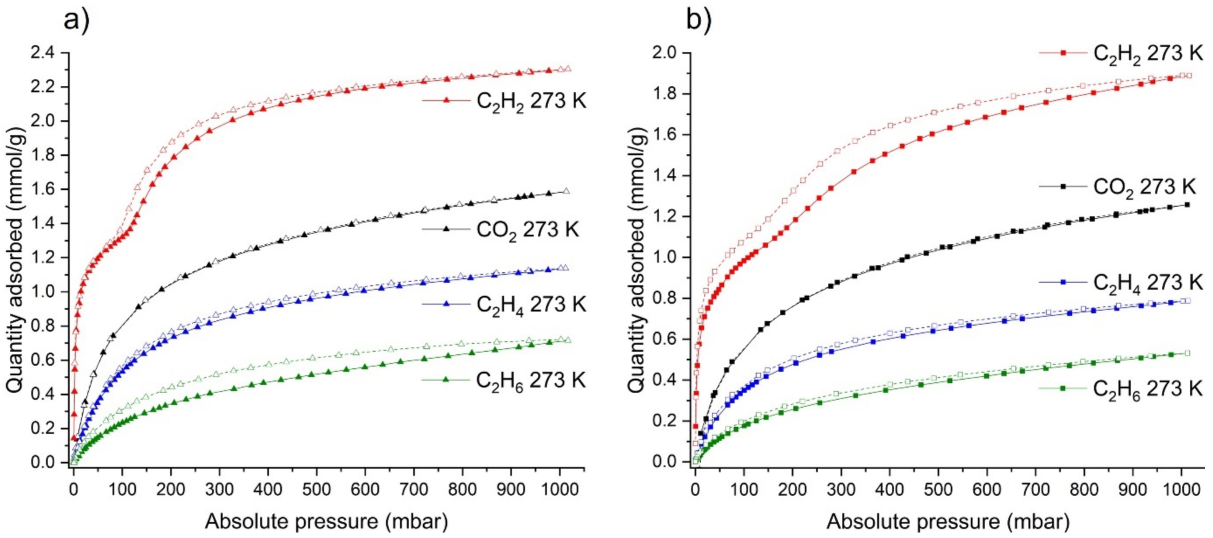

Figure S22: C_2H_2, CO_2, C_2H_4, and C_2H_6 isotherms at 273 K for a) **2** – Layering and b) **2** – Slurrying.

4.6 PXRD after thermal decomposition of 2

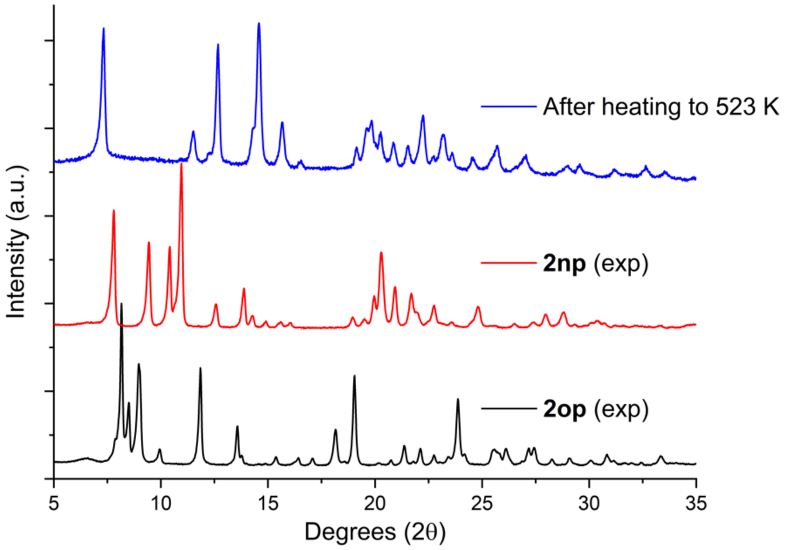

Figure S23: An overlay of the experimental PXRD of **2** collected after heating a sample to 523 K. As seen in the TGA and DSC plots, the material thermally decomposes at this temperature and so the PXRD of the material changes. Experimental PXRD patterns of **2op** and **2np** are shown for comparison.

4.7 IAST Selectivity Calculations

The selectivities for C_2H_2/CO_2 and C_2H_2/C_2H_4 gas mixtures of **2np** were calculated from single component adsorption isotherms using the Ideal Adsorbed Solution Theory (IAST)[30, 31] using the software IAST++.[32] Single-component adsorption isotherms for each gas at 298 K were fitted to the dual-site Langmuir equation:

$$n(P) = \frac{q_1(k_1P)}{1+(k_1P)} + \frac{q_2(k_2P)}{1+(k_2P)}$$

Where P is the total pressure (mbar) of the bulk gas at equilibrium with the adsorbed phase, q_1 and q_2 are the saturation uptakes (in mmol//g) for sites 1 and 2, respectively, k_1 and k_2 are the affinity coefficients (in $mbar^{-1}$) for sites 1 and 2, respectively, and n(P) is the uptake (mmol/g) as a function of pressure. After the isotherms have been parameterized, mixed-gas fractional uptakes are calculated and the IAST selectivity ($S_{i/j}$) is obtained through the equation:

$$S_{i/j} = \frac{(\frac{x_i}{x_j})}{(\frac{y_i}{y_j})}$$

Where $x_i$and x_j are the mole fractions of components i and j, respectively, in the adsorbed phase, and y_i and y_j are the mole fractions of components i and j, respectively, in the gas phase.

Dual-site Langmuir parameter for C_2H_2/CO_2 and C_2H_2/C_2H_4 gas mixtures are listed in Table S6.

Table S7. Isotherm fitting parameters and R^2 values for IAST calculations.

Adsorbent	Adsorbate	Model	R^2	q_1 (mmol/g)	k_1 ($mbar^{-1}$)	q_2 (mmol/g)	k_2 ($mbar^{-1}$)
**2np – Layering**	C_2H_2 298 K[a]	Dual-site Langmuir	0.99994	1.15754	0.0806603	172.415	0.00000355849
	CO_2 298 K	Dual-site Langmuir	0.999998	1.19523	0.00378738	7.33412	0.0000236861
	C_2H_4 298 K[a]	Dual-site Langmuir	0.99999	0.765195	0.00337103	7.52541	0.0000408504
**2np – Slurrying**	C_2H_2 298 K[a]	Dual-site Langmuir	0.999983	0.689786	0.0841912	0.917575	0.00149274
	CO_2 298 K	Dual-site Langmuir	0.999998	0.278837	0.00936367	1.31633	0.00113223
	C_2H_4 298 K[a]	Dual-site Langmuir	0.999992	0.328575	0.00507569	0.890057	0.00047351

Note, data truncated at the emergence of flexibility for C_2H_2 at 0.328 mbar for layering[a] or 0.427 mbar for slurrying[b].

4.8 Dynamic Column Breakthrough (DCB) experiments

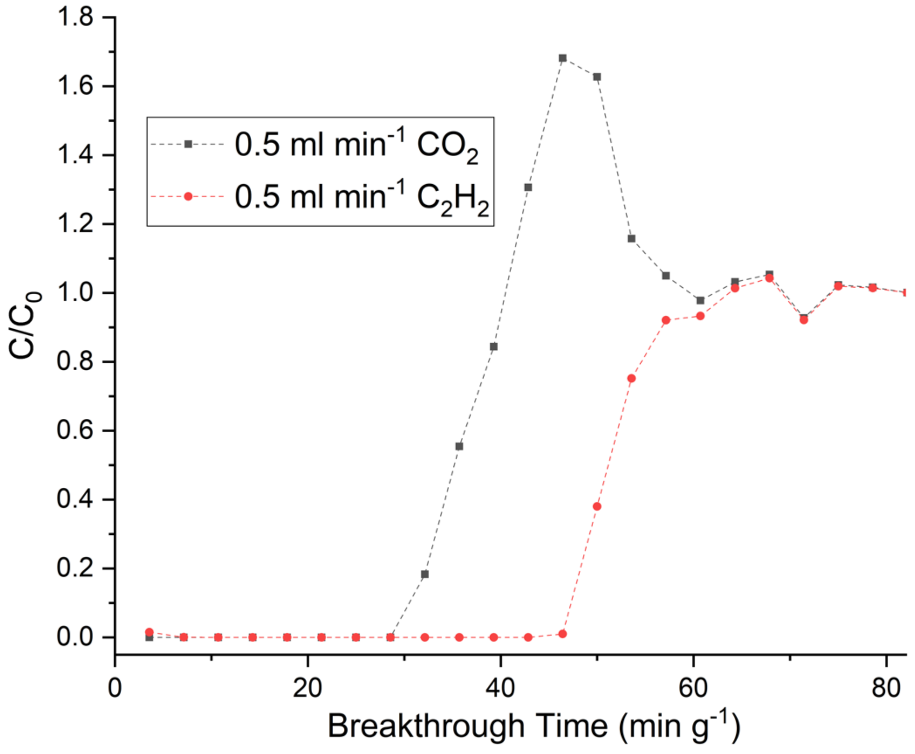

Figure S24: DCB experiment of a slurry-synthesized sample of **2** for a 1:1 C_2H_2/CO_2 gas mixture (total gas flow of 1 cm^3 min^{-1}). Breakthrough time normalized for sample mass (1.4 g).

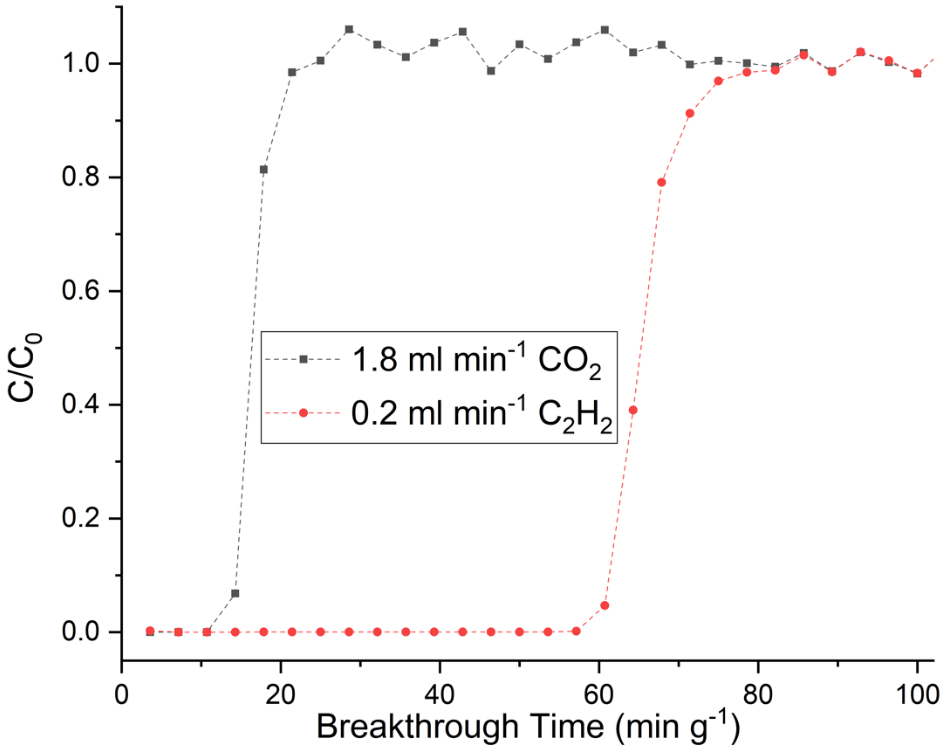

Figure S25: DCB experiment of a slurry-synthesized sample of **2** for a 1:9 C_2H_2/CO_2 gas mixture (total gas flow of 2 cm^3 min^{-1}). Breakthrough time normalized for sample mass (1.4 g).

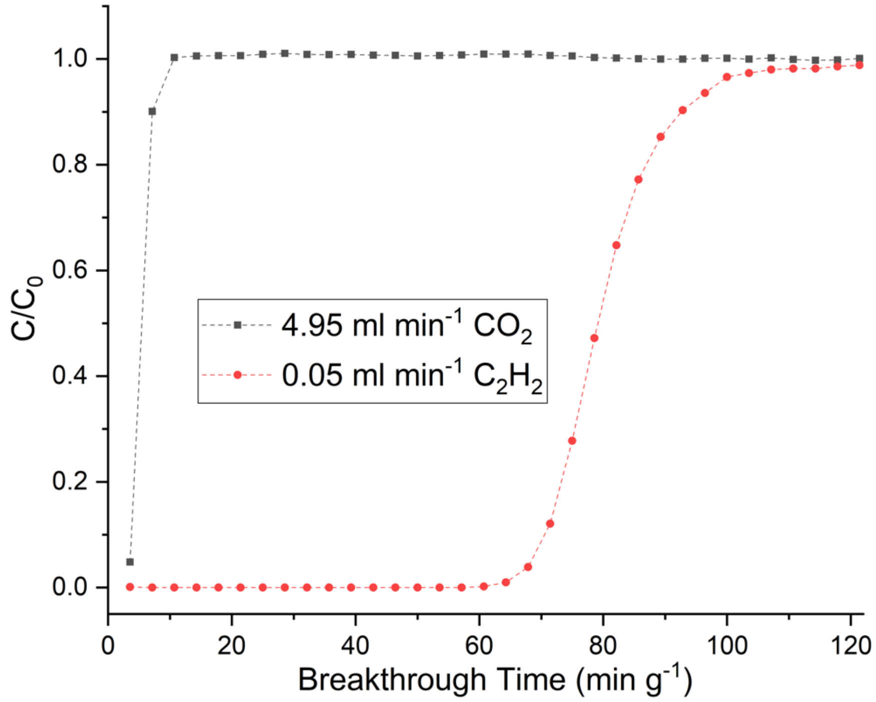

Figure S26: DCB experiment of a slurry-synthesized sample of **2** for a 1:99 C_2H_2/CO_2 gas mixture (total gas flow of 5 cm^3 min^{-1}). Breakthrough time normalized for sample mass (1.4 g).

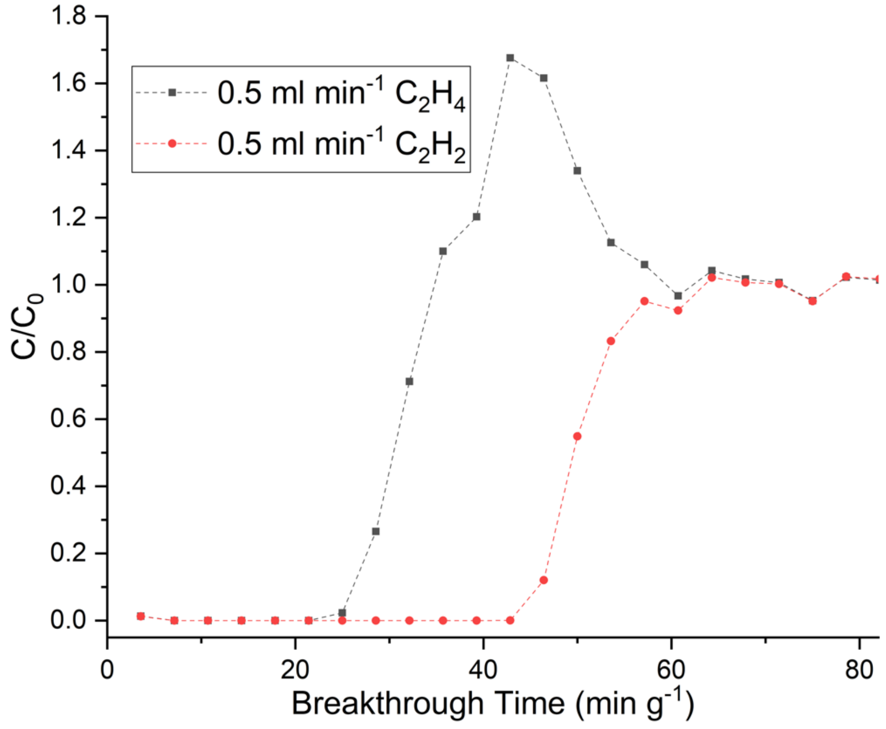

Figure S27: DCB experiment of a slurry-synthesized sample of **2** for a 1:1 C_2H_2/C_2H_4 gas mixture (total gas flow of 1 cm^3 min^{-1}). Breakthrough time normalized for sample mass (1.4 g).

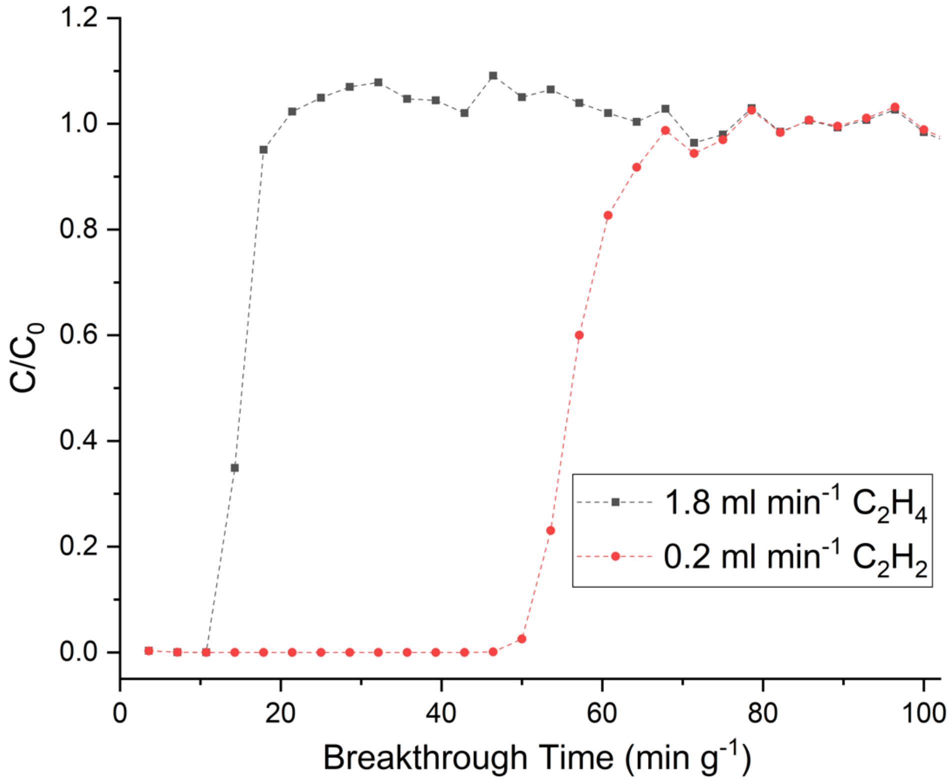

Figure S28: DCB experiment of a slurry-synthesized sample of **2** for a 1:9 C_2H_2/C_2H_4 gas mixture (total gas flow of 2 cm^3 min^{-1}). Breakthrough time normalized for sample mass (1.4 g).

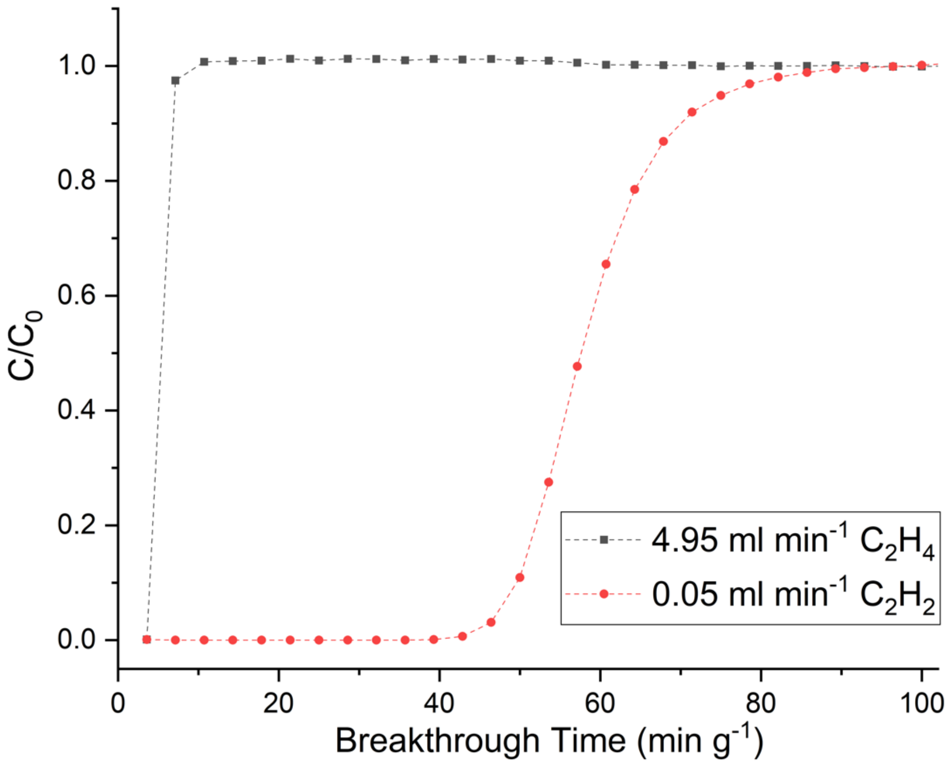

Figure S29: DCB experiment of a slurry-synthesized sample of **2** for a 1:99 C_2H_2/C_2H_4 gas mixture (total gas flow of 5 cm^3 min^{-1}). Breakthrough time normalized for sample mass (1.4 g).

4.9 Gas sorption comparison with leading materials

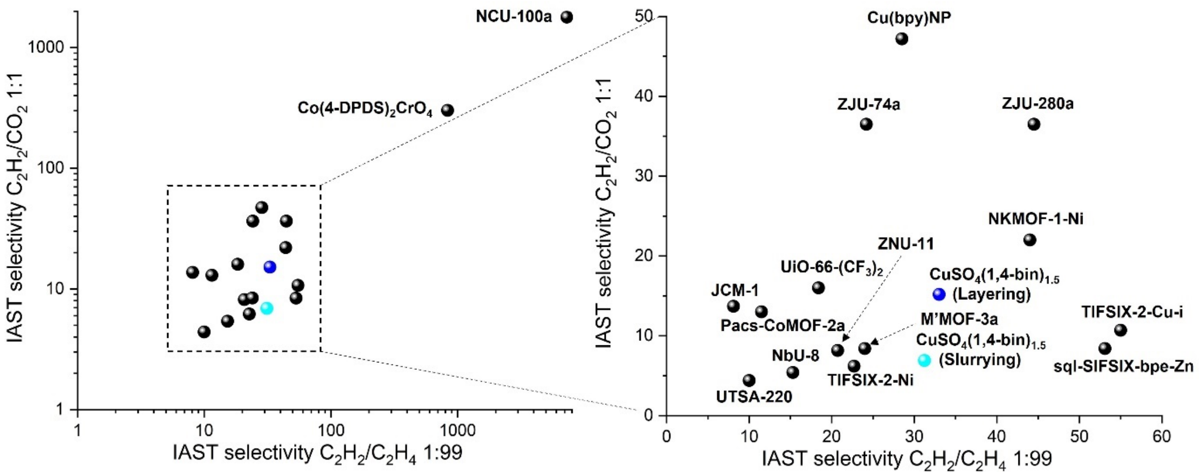

Figure S30: Scatter plot of 1:99 C_2H_2/C_2H_4 and 1:1 C_2H_2/CO_2 IAST selectivity plots of leading materials, for which both values are reported. For more information, see Table S7.

Table S8. Comparison of adsorption capacities and IAST selectivity with C_2H_2/CO_2 1:1 and C_2H_2/C_2H_4 1:99 of $CuSO_4(1,4\text{-bin})_{1.5}$ and other high performing materials at 298 K and 1.0 bar (unless otherwise stated).

Name	C_2H_2 uptake ($cm^3\ g^{-1}$)	C_2H_4 uptake ($cm^3\ g^{-1}$)	CO_2 uptake ($cm^3\ g^{-1}$)	C_2H_2/CO_2 1:1	C_2H_2/C_2H_4 1:99	ref
SOFOUR-TEPE-Zn	69.4	-	81.0	16833	-	33
NCU-100a	102.4	7.2	11	1786.6	7291	34
SIFSIX-14-Cu-i (UTSA-200a)	85.0	20.0	-	-	6320	35
ZJU-300a	120.3^a	53.5^a	-	-	1672^a	36
GeFSIX-14-Cu-i (ZU-33)	84.6^d	43.7^d	-	-	>1100	37
Co(4-DPDS)$_2$CrO$_4$	54.4	4.9	9.0	302	834	38
TIFSIX-14-Cu-i (ZU-13)	84.7^d	31.6^d	-	-	229	39
ZUL-100	161.1	61.8	-	-	175	40
Ni$_3$(pzdc)$_2$(7 Hade)$_2$	52.9	33.1	-	-	168	41
ZUL-300	122.3	56.0	-	-	139	42
ZUL-200	153.4	44.6	-	-	114	40
GEFSIX-2-Cu-i (ZU-32)	88.9^d	49.0^d	-	-	67	37
TIFSIX-2-Cu-i	91.8	58.2	96.3	10.7	55	43, 44
sql-SIFSIX-bpe-Zn	40	28	40	8.4	53.1	45
SIFSIX-2-Cu-i	90.0	49.1	-	-	44.54	46
ZJU-280a	106.2^a	66.1^a	71.0^a	18.1^a	44.5^a	47
NKMOF-1-Ni	61.0	46.8	51.1	22.0	44.0	48
NbOFFIVE-2-Ni-i (ZU-62-Ni)	67.4	17.9	-	-	37.2	49
**CuSO$_4$(1,4-bin)$_{1.5}$ (Layering)**	**51.1**	**19.9**	**11.6**	**15.18**	**33.01**	**This work**
**CuSO$_4$(1,4-bin)$_{1.5}$ (Slurrying)**	**31.9**	**12.6**	**21.4**	**6.91**	**31.24**	**This work**
NbOFFIVE-2-Cu-i (ZU-62)	84.1^d	52.4^d	99.9	-	29	39, 50
Cu(bpy)NP	50.7	40.8	25.1	-	28.5	51
ZJU-74a	85.7^a	71.0^a	70.1^a	36.5^a	24.2^a	52
M'MOF-3a	42.6^b	9.0^b	13^b	8.41^b	24.03^b	53, 54
TIFSIX-2-Ni (ZU-12-Ni)	94.3	54.2	101.7	6.2^e	22.7	55
NbOFFIVE-4-Cu (ZNU-11)	45.5	14.2	22.5	8.17	20.73	56
UiO-66-(CF$_3$)$_2$	67.0	17.6	15.3	16.0	18.4	57
TIFSIX-3-Co (ZU-16-Co)	93.6	65.4	-	-	17	58
NbU-8	190.0^c	114.9^c	56.9^c	5.4^c	15.3^c	59
Pacs-CoMOF-2a	121	63	66	13.0	11.5	60
UTSA-100a	95.6^a	37.2^a	-	-	10.72^a	61
SIFSIX-1-Cu	190.4	92.1	-	-	10.63	46
UTSA-220	76.2	56.7	75.7	4.4	10	62
SIFSIX-3-Zn	81.5	50.2	-	-	8.82	46
SIFSIX-2-Cu	120.5	45.2	-	-	6.0	46
SIFSIX-3-Ni	73.9	44.4	60.5	-	5.03	43, 44
JCM-1	75	35	38	13.7	8.1	63
SOFOUR-1-Zn	69.4	-	80.6	6.6	-	64

^{a}296 K; ^{b}295 K; ^{c}293 K; ^{d}1 atm; ^{e} C_2H_2/CO_2 2:1.

5 References

1. Altman, R. A.; Buchwald, S. L., 4,7-Dimethoxy-1,10-phenanthroline: An Excellent Ligand for the Cu-Catalyzed N-Arylation of Imidazoles. *Organic Letters* **2006,** *8* (13), 2779-2782.
2. Roy, M.; Pham, D. N. K.; Kreider-Mueller, A.; Golen, J. A.; Manke, D. R., First-row transition metal-pyridine (py)-sulfate [(py)xM](SO4) complexes (M = Ni, Cu and Zn): crystal field theory in action. *Acta Crystallographica Section C* **2018,** *74* (3), 263-268.
3. Degen, T.; Sadki, M.; Bron, E.; König, U.; Nénert, G., The HighScore suite. *Powder Diffraction* **2014,** *29* (S2), S13-S18.
4. Macrae, C. F.; Bruno, I. J.; Chisholm, J. A.; Edgington, P. R.; McCabe, P.; Pidcock, E.; Rodriguez-Monge, L.; Taylor, R.; van de Streek, J.; Wood, P. A., Mercury CSD 2.0 - new features for the visualization and investigation of crystal structures. *Journal of Applied Crystallography* **2008,** *41* (2), 466-470.
5. APEX4. Ver. 2021.4-0. Bruker AXS Inc., M., Wisconsin, USA, 2021.
6. Krause, L.; Herbst-Irmer, R.; Sheldrick, G. M.; Stalke, D., Comparison of Silver and Molybdenum Microfocus X-ray Sources for Single Crystal Structure Determination. *Journal of Applied Crystallography* **2015,** *48*, 3-10.
7. XPREP Ver. **2014**/2, B. A. I., Madison, Wisconsin, USA, 2014.
8. Dolomanov, O. V.; Bourhis, L. J.; Gildea, R. J.; Howard, J. A. K.; Puschmann, H., OLEX2: a complete structure solution, refinement and analysis program. *Journal of Applied Crystallography* **2009,** *42* (2), 339-341.
9. Sheldrick, G., SHELXT - Integrated space-group and crystal-structure determination. *Acta Crystallographica, Section A: Foundations and Advances* **2015,** *71* (1), 3-8.
10. Sheldrick, G., Crystal Structure Refinement with SHELXL. *Acta Crystallographica, Section C: Structural Chemistry* **2015,** *71*, 3-8.
11. Spek, A. L., PLATON SQUEEZE: a tool for the calculation of the disordered solvent contribution to the calculated structure factors. *Acta Crystallographica, Section C: Structural Chemistry* **2015,** *71* (Pt 1), 9-18.
12. Revenko, M. D.; Bourosh, P. N.; Stratulat, E. F.; Gdaniec, M.; Lipkowski, Y.; Korzha, I. D.; Simonov, Y. A., Synthesis and structure of copper(II) coordination compounds with 8-quinolinecarboxaldehyde thio- and 4-phenylthiosemicarbazones. *Russian Journal of Inorganic Chemistry* **2010,** *55* (9), 1387-1397.
13. Foxon, Simon P.; Torres, Gemma R.; Walter, O.; Pedersen, Jens Z.; Toftlund, H.; Hüber, M.; Falk, K.; Haase, W.; Cano, J.; Lloret, F.; Julve, M.; Schindler, S., Syntheses, Structures, and Magnetic Properties of Copper(II) Complexes with 1,3-[Bis(2-pyridylmethyl)amino]benzene (1,3-tpbd) as Ligand. *European Journal of Inorganic Chemistry* **2004,** *2004* (2), 335-343.
14. Bingham, A. G.; Bögge, H.; Müller, A.; Ainscough, E. W.; Brodie, A. M., Synthetic, spectroscopic, and X-ray crystallographic studies on binuclear copper(II) complexes with a tridentate NNS-bonding 2-formylpyridine thiosemicarbazone ligand. The characterization of both neutral and deprotonated co-ordinated ligand structures. *Journal of the Chemical Society, Dalton Transactions* **1987,** (3), 493-499.
15. Wang, D.-Z.; Li, J.-P.; Fan, J.-Z.; Jia, D.-Z., Metal–organic coordination architectures of bis(N-imidazolyl) pyridazine: Syntheses, structures, emission and photocatalytic properties. *Polyhedron* **2016,** *111*, 123-131.
16. Zhao, W.; Fan, J.; Song, Y.; Kawaguchi, H.; Okamura, T.-a.; Sun, W.-Y.; Ueyama, N., Syntheses, crystal structures and properties of novel copper(ii) complexes obtained by reactions of copper(ii) sulfate pentahydrate with tripodal ligands. *Dalton Transactions* **2005,** (8), 1509-1517.
17. Bartholomä, M.; Ploier, B.; Cheung, H.; Ouellette, W.; Zubieta, J., Coordination polymers of Cu(II) and Cd(II) with bifunctional chelates of the type dipicolylamino-alkylcarboxylate, (NC5H4CH2)2N(CH2)nCO2H (n = 1, 2, 3 and 4). *Inorganica Chimica Acta* **2010,** *363* (8), 1659-1665.
18. Bocian, A.; Gorczyński, A.; Marcinkowski, D.; Witomska, S.; Kubicki, M.; Mech, P.; Bogunia, M.; Brzeski, J.; Makowski, M.; Pawluć, P.; Patroniak, V., New benzothiazole based copper(II)

hydrazone Schiff base complexes for selective and environmentally friendly oxidation of benzylic alcohols: The importance of the bimetallic species tuned by the choice of the counterion. *Journal of Molecular Liquids* **2020,** *302*, 112590.
19. Bryant, M. R.; Burrows, A. D.; Fitchett, C. M.; Hawes, C. S.; Hunter, S. O.; Keenan, L. L.; Kelly, D. J.; Kruger, P. E.; Mahon, M. F.; Richardson, C., The synthesis and characterisation of coordination and hydrogen-bonded networks based on 4-(3,5-dimethyl-1H-pyrazol-4-yl)benzoic acid. *Dalton Transactions* **2015,** *44* (19), 9269-9280.
20. Zimmerman, J. R.; de Bettencourt-Dias, A., Homobinuclear sulfato-bridged and mononuclear nitrato complexes of Cu(II) with thiophen-2-yl-dipicolylamine; structure and anion-dependent absorption spectra. *Inorganic Chemistry Communications* **2011,** *14* (5), 753-758.
21. Xie, J.; Chen, X.; Liu, G. X.; Sun, W. Y., Synthesis and crystal structure of Cu(II) complex with two-dimensional network structure. *Chinese Journal of Inorganic Chemistry* **2007,** *23* (7), 1295-1298.
22. Patel, A. K.; Jadeja, R. N.; Roy, H.; Patel, R. N.; Patel, S. K.; Butcher, R. J.; Cortijo, M.; Herrero, S., Copper(II) hydrazone complexes with different nuclearities and geometries: Synthesis, structural characterization, antioxidant SOD activity and antiproliferative properties. *Polyhedron* **2020,** *186*, 114624.
23. Soek, R. N.; da C. Gouveia, T. L.; Garbelini, E. R.; dos R. Crespan, E.; Pineider, F.; Poneti, G.; Machado, G. S.; Ribeiro, R. R.; Hörner, M.; Nunes, F. S., Structural, Magnetic, Spectroscopic and Density Functional Theory (DFT) Analysis of Bis((1-((E)-2-pyridinylmethylidene)semicarbazone)copper(II)sulfate) Dihydrate Complex. *ChemistrySelect* **2017,** *2* (27), 8451-8458.
24. Almesåker, A.; Bourne, S. A.; Ramon, G.; Scott, J. L.; Strauss, C. R., Coordination chemistry of N,N,4-tris(pyridin-2-ylmethyl)aniline: a novel flexible, multimodal ligand. *CrystEngComm* **2007,** *9* (11), 997-1010.
25. Pham, D. N. K.; Roy, M.; Kreider-Mueller, A.; Golen, J. A.; Manke, D. R., More crystal field theory in action: the metal-4-picoline (pic)-sulfate [M(pic)x]SO4 complexes (M = Fe, Co, Ni, Cu, Zn, and Cd). *Acta Crystallographica, Section C: Structural Chemistry* **2019,** *75* (5), 568-574.
26. de Bettencourt-Dias, A.; Scott, V. J.; Hugdal, S., Counter-anions and their coordination behavior with Cu(II) complexes of thiophen-3-yl-dipicolylamine. *Inorganica Chimica Acta* **2010,** *363* (14), 4088-4095.
27. D'Alessio, D.; Lombardo, D. M.; Vaughan, J. G.; Skelton, B. W.; Barnard, K. R.; Ogden, M. I., Structural variations in metal complexes of a tertiary α-hydroxyoxime. *Dalton Transactions* **2015,** *44* (16), 7163-7168.
28. Qin, S.; Nie, C.; Weng, Z.; Chen, Z.; Liang, F., Three Copper(II) Complexes of a “Tritopic” Hydrazone Ligand: Synthesis and Structural Characterization. *Zeitschrift für anorganische und allgemeine Chemie* **2011,** *637* (14-15), 2294-2299.
29. Li, M.-X.; Miao, Z.-X.; Shao, M.; Liang, S.-W.; Zhu, S.-R., Metal-Organic Frameworks Constructed from 2,4,6-Tris(4-pyridyl)-1,3,5-triazine. *Inorganic Chemistry* **2008,** *47* (11), 4481-4489.
30. Myers, A. L.; Prausnitz, J. M., Thermodynamics of mixed-gas adsorption. *AIChE Journal* **1965,** *11* (1), 121-127.
31. Walton, K. S.; Sholl, D. S., Predicting multicomponent adsorption: 50 years of the ideal adsorbed solution theory. *AIChE Journal* **2015,** *61* (9), 2757-2762.
32. Lee, S.; Lee, J. H.; Kim, J., User-friendly graphical user interface software for ideal adsorbed solution theory calculations. *Korean Journal of Chemical Engineering* **2018,** *35* (1), 214-221.
33. Liu, X.; Zhang, P.; Xiong, H.; Zhang, Y.; Wu, K.; Liu, J.; Krishna, R.; Chen, J.; Chen, S.; Zeng, Z.; Deng, S.; Wang, J., Engineering Pore Environments of Sulfate-Pillared Metal-Organic Framework for Efficient C2H2/CO2 Separation with Record Selectivity. *Advanced Materials* **2023,** *35* (20), 2210415.
34. Wang, J.; Zhang, Y.; Zhang, P.; Hu, J.; Lin, R.-B.; Deng, Q.; Zeng, Z.; Xing, H.; Deng, S.; Chen, B., Optimizing Pore Space for Flexible-Robust Metal–Organic Framework to Boost Trace Acetylene Removal. *Journal of the American Chemical Society* **2020,** *142* (21), 9744-9751.

35. Li, B.; Cui, X.; O'Nolan, D.; Wen, H.-M.; Jiang, M.; Krishna, R.; Wu, H.; Lin, R.-B.; Chen, Y.-S.; Yuan, D.; Xing, H.; Zhou, W.; Ren, Q.; Qian, G.; Zaworotko, M. J.; Chen, B., An Ideal Molecular Sieve for Acetylene Removal from Ethylene with Record Selectivity and Productivity. *Advanced Materials* **2017,** *29* (47), 1704210.
36. Gu, X.-W.; Wu, E.; Wang, J.-X.; Wen, H.-M.; Chen, B.; Li, B.; Qian, G., Programmed fluorine binding engineering in anion-pillared metal-organic framework for record trace acetylene capture from ethylene. *Science Advances* **2023,** *9* (31), eadh0135.
37. Zhang, Z.; Cui, X.; Yang, L.; Cui, J.; Bao, Z.; Yang, Q.; Xing, H., Hexafluorogermanate (GeFSIX) Anion-Functionalized Hybrid Ultramicroporous Materials for Efficiently Trapping Acetylene from Ethylene. *Industrial & Engineering Chemistry Research* **2018,** *57* (21), 7266-7274.
38. Zheng, F.; Chen, R.; Liu, Y.; Yang, Q.; Zhang, Z.; Yang, Y.; Ren, Q.; Bao, Z., Strengthening Intraframework Interaction within Flexible MOFs Demonstrates Simultaneous Sieving Acetylene from Ethylene and Carbon Dioxide. *Advanced Science* **2023,** *10* (9), 2207127.
39. O'Nolan, D.; Kumar, A.; Chen, K.-J.; Mukherjee, S.; Madden, D. G.; Zaworotko, M. J., Finding the Optimal Balance between the Pore Size and Pore Chemistry in Hybrid Ultramicroporous Materials for Trace Acetylene Capture. *ACS Applied Nano Materials* **2018,** *1* (11), 6000-6004.
40. Shen, J.; He, X.; Ke, T.; Krishna, R.; van Baten, J. M.; Chen, R.; Bao, Z.; Xing, H.; Dincă, M.; Zhang, Z.; Yang, Q.; Ren, Q., Simultaneous interlayer and intralayer space control in two-dimensional metal−organic frameworks for acetylene/ethylene separation. *Nature Communications* **2020,** *11* (1), 6259.
41. Zhang, Z.; Peh, S. B.; Wang, Y.; Kang, C.; Fan, W.; Zhao, D., Efficient Trapping of Trace Acetylene from Ethylene in an Ultramicroporous Metal–Organic Framework: Synergistic Effect of High-Density Open Metal and Electronegative Sites. *Angewandte Chemie International Edition* **2020,** *59* (43), 18927-18932.
42. Song, Y.; Ke, T.; Shen, J.; Li, J.; Zhu, X.; Yang, L.; Zhang, Z.; Bao, Z.; Ren, Q.; Yang, Q., Shaped layered two-dimensional fluorinated metal-organic frameworks for highly efficient acetylene/ethylene separation. *Separation and Purification Technology* **2023,** *323*, 124377.
43. Chen, K.-J.; Scott, Hayley S.; Madden, David G.; Pham, T.; Kumar, A.; Bajpai, A.; Lusi, M.; Forrest, Katherine A.; Space, B.; Perry, John J.; Zaworotko, Michael J., Benchmark C2H2/CO2 and CO2/C2H2 Separation by Two Closely Related Hybrid Ultramicroporous Materials. *Chem* **2016,** *1* (5), 753-765.
44. Chen, K.-J.; Madden, D. G.; Mukherjee, S.; Pham, T.; Forrest, K. A.; Kumar, A.; Space, B.; Kong, J.; Zhang, Q.-Y.; Zaworotko, M. J., Synergistic sorbent separation for one-step ethylene purification from a four-component mixture. *Science* **2019,** *366* (6462), 241-246.
45. Shivanna, M.; Otake, K.-i.; Song, B.-Q.; van Wyk, L. M.; Yang, Q.-Y.; Kumar, N.; Feldmann, W. K.; Pham, T.; Suepaul, S.; Space, B.; Barbour, L. J.; Kitagawa, S.; Zaworotko, M. J., Benchmark Acetylene Binding Affinity and Separation through Induced Fit in a Flexible Hybrid Ultramicroporous Material. *Angewandte Chemie International Edition* **2021,** *60* (37), 20383-20390.
46. Cui, X.; Chen, K.; Xing, H.; Yang, Q.; Krishna, R.; Bao, Z.; Wu, H.; Zhou, W.; Dong, X.; Han, Y.; Li, B.; Ren, Q.; Zaworotko, M. J.; Chen, B., Pore chemistry and size control in hybrid porous materials for acetylene capture from ethylene. *Science* **2016,** *353* (6295), 141-144.
47. Qian, Q.-L.; Gu, X.-W.; Pei, J.; Wen, H.-M.; Wu, H.; Zhou, W.; Li, B.; Qian, G., A novel anion-pillared metal–organic framework for highly efficient separation of acetylene from ethylene and carbon dioxide. *Journal of Materials Chemistry A* **2021,** *9* (14), 9248-9255.
48. Peng, Y.-L.; Pham, T.; Li, P.; Wang, T.; Chen, Y.; Chen, K.-J.; Forrest, K. A.; Space, B.; Cheng, P.; Zaworotko, M. J.; Zhang, Z., Robust Ultramicroporous Metal–Organic Frameworks with Benchmark Affinity for Acetylene. *Angewandte Chemie International Edition* **2018,** *57* (34), 10971-10975.
49. Yang, L.; Jin, A.; Ge, L.; Cui, X.; Xing, H., A novel interpenetrated anion-pillared porous material with high water tolerance afforded efficient C2H2/C2H4 separation. *Chemical Communications* **2019,** *55* (34), 5001-5004.

50. Hou, W.; Cheng, J.; Hu, L.; Wu, Y.; Zhou, J., Mixed matrix membranes based on NbOF52− anion-pillared porous MOFs for efficient CO2 separation. *Journal of Membrane Science* **2024,** *693*, 122323.
51. Liu, Y.; Liu, J.; Xiong, H.; Chen, J.; Chen, S.; Zeng, Z.; Deng, S.; Wang, J., Negative electrostatic potentials in a Hofmann-type metal-organic framework for efficient acetylene separation. *Nature Communications* **2022,** *13* (1), 5515.
52. Pei, J.; Shao, K.; Wang, J.-X.; Wen, H.-M.; Yang, Y.; Cui, Y.; Krishna, R.; Li, B.; Qian, G., A Chemically Stable Hofmann-Type Metal–Organic Framework with Sandwich-Like Binding Sites for Benchmark Acetylene Capture. *Advanced Materials* **2020,** *32* (24), 1908275.
53. He, Y.; Krishna, R.; Chen, B., Metal–organic frameworks with potential for energy-efficient adsorptive separation of light hydrocarbons. *Energy & Environmental Science* **2012,** *5* (10), 9107-9120.
54. Xiang, S.-C.; Zhang, Z.; Zhao, C.-G.; Hong, K.; Zhao, X.; Ding, D.-R.; Xie, M.-H.; Wu, C.-D.; Das, M. C.; Gill, R.; Thomas, K. M.; Chen, B., Rationally tuned micropores within enantiopure metal-organic frameworks for highly selective separation of acetylene and ethylene. *Nature Communications* **2011,** *2* (1), 204.
55. Jiang, M.; Cui, X.; Yang, L.; Yang, Q.; Zhang, Z.; Yang, Y.; Xing, H., A thermostable anion-pillared metal-organic framework for C2H2/C2H4 and C2H2/CO2 separations. *Chemical Engineering Journal* **2018,** *352*, 803-810.
56. Han, Y.; Jiang, Y.; Hu, J.; Wang, L.; Zhang, Y., Efficient C2H2/CO2 and C2H2/C2H4 separations in a novel fluorinated metal–organic framework. *Separation and Purification Technology* **2024,** *332*, 125777.
57. Chen, Y.; Xiong, Q.; Wang, Y.; Du, Y.; Wang, Y.; Yang, J.; Li, L.; Li, J., Boosting molecular recognition of acetylene in UiO-66 framework through pore environment functionalization. *Chemical Engineering Science* **2021,** *237*, 116572.
58. Zhang, Z.; Ding, Q.; Cui, J.; Cui, X.; Xing, H., Fine-Tuning Pore Dimension in Hybrid Ultramicroporous Materials Boosting Simultaneous Trapping of Trace Alkynes from Alkenes. *Small* **2020,** *16* (49), 2005360.
59. Li, Q.; Wu, N.; Li, J.; Wu, D., A Highly Connected Trinuclear Cluster Based Metal–Organic Framework for Efficient Separation of C2H2/C2H4 and C2H2/CO2. *Inorganic Chemistry* **2020,** *59* (18), 13005-13008.
60. Chen, D.-M.; Sun, C.-X.; Zhang, N.-N.; Si, H.-H.; Liu, C.-S.; Du, M., Tunable Robust pacs-MOFs: a Platform for Systematic Enhancement of the C2H2 Uptake and C2H2/C2H4 Separation Performance. *Inorganic Chemistry* **2018,** *57* (5), 2883-2889.
61. Hu, T.-L.; Wang, H.; Li, B.; Krishna, R.; Wu, H.; Zhou, W.; Zhao, Y.; Han, Y.; Wang, X.; Zhu, W.; Yao, Z.; Xiang, S.; Chen, B., Microporous metal–organic framework with dual functionalities for highly efficient removal of acetylene from ethylene/acetylene mixtures. *Nature Communications* **2015,** *6* (1), 7328.
62. Li, H.; Li, L.; Lin, R.-B.; Ramirez, G.; Zhou, W.; Krishna, R.; Zhang, Z.; Xiang, S.; Chen, B., Microporous Metal–Organic Framework with Dual Functionalities for Efficient Separation of Acetylene from Light Hydrocarbon Mixtures. *ACS Sustainable Chemistry & Engineering* **2019,** *7* (5), 4897-4902.
63. Lee, J.; Chuah, C. Y.; Kim, J.; Kim, Y.; Ko, N.; Seo, Y.; Kim, K.; Bae, T. H.; Lee, E., Separation of Acetylene from Carbon Dioxide and Ethylene by a Water-Stable Microporous Metal–Organic Framework with Aligned Imidazolium Groups inside the Channels. *Angewandte Chemie International Edition* **2018,** *57* (26), 7869-7873.
64. Sensharma, D.; O'Hearn, D. J.; Koochaki, A.; Bezrukov, A. A.; Kumar, N.; Wilson, B. H.; Vandichel, M.; Zaworotko, M. J., The First Sulfate-Pillared Hybrid Ultramicroporous Material, SOFOUR-1-Zn, and Its Acetylene Capture Properties. *Angewandte Chemie International Edition* **2022,** *61* (8), e202116145.
